# TAM receptors mediate the Fpr2-driven pain resolution and fibrinolysis after nerve injury

**DOI:** 10.1007/s00401-024-02840-9

**Published:** 2024-12-16

**Authors:** Beate Hartmannsberger, Adel Ben-Kraiem, Sofia Kramer, Carolina Guidolin, Ida Kazerani, Kathrin Doppler, Dominique Thomas, Robert Gurke, Marco Sisignano, Pranav P. Kalelkar, Andrés J. García, Paula V. Monje, Michael Sammeth, Asma Nusrat, Alexander Brack, Susanne M. Krug, Claudia Sommer, Heike L. Rittner

**Affiliations:** 1 Centre for Interdisciplinary Pain Medicine, Department of Anaesthesiology, Intensive Care, Emergency and Pain Medicine, University Hospital Würzburg, Würzburg, Germany; 2 Helmholtz Institute for Metabolic, Obesity and Vascular Research, Diet-Induced Metabolic Alterations Group, Leipzig, Germany; 3 Department of Neurology, University Hospital Würzburg, Würzburg, Germany; 4 Goethe University, Frankfurt, Faculty of Medicine, Institute of Clinical Pharmacology, Frankfurt Am Main, Germany; 5 Fraunhofer Institute for Translational Medicine and Pharmacology ITMP, and Fraunhofer Cluster of Excellence of Immune Mediate Diseases CIMD, Frankfurt Am Main, Germany; 6 George W. Woodruff School of Mechanical Engineering, Petit Institute for Bioengineering and Bioscience, Georgia Institute of Technology, Atlanta, USA; 7 Department of Neurosurgery, College of Medicine, University of Kentucky, Lexington, KY USA; 8 Department of Applied Sciences and Health, Coburg University of Applied Sciences and Art, Coburg, Germany; 9 Department of Anaesthesiology, Intensive Care, Emergency and Pain Medicine, University Hospital Würzburg, Würzburg, Germany; 10 Department of Pathology, University of Michigan, Ann Arbor, MI USA; 11 Charité-Universitätsmedizin Berlin, Clinical Physiology/Nutritional Medicine, Berlin, Germany

**Keywords:** Blood–nerve barrier, Fibrinogen, Pain resolution, Resolvin D1, Chronic constriction injury, TAM receptors, Nanoparticles

## Abstract

Nerve injury causes neuropathic pain and multilevel nerve barrier disruption. Nerve barriers consist of perineurial, endothelial and myelin barriers. So far, it is unclear whether resealing nerve barriers fosters pain resolution and recovery. To this end, we analysed the nerve barrier property portfolio, pain behaviour battery and lipidomics for precursors of specialized pro-resolving meditators (SPMs) and their receptors in chronic constriction injury of the rat sciatic nerve to identify targets for pain resolution by resealing the selected nerve barriers. Of the three nerve barriers—perineurium, capillaries and myelin—only capillary tightness specifically against larger molecules, such as fibrinogen, recuperated with pain resolution. Fibrinogen immunoreactivity was elevated in rats not only at the time of neuropathic pain but also in nerve biopsies from patients with (but not without) painful polyneuropathy, indicating that sealing of the vascular barrier might be a novel approach in pain treatment. Hydroxyeicosatetraenoic acid (15R-HETE), a precursor of aspirin-triggered lipoxin A4, was specifically upregulated at the beginning of pain resolution. Repeated local application of resolvin D1-laden nanoparticles or Fpr2 agonists sex-independently resulted in accelerated pain resolution and fibrinogen removal. Clearing macrophages (*Cd206)* were boosted and fibrinolytic pathways (*Plat)* were induced, while inflammation (*Tnfα)* and inflammasomes (*Nlrp3)* were unaffected by this treatment. Blocking TAM receptors (Tyro3, Axl and Mer) and tyrosine kinase receptors linking haemostasis and inflammation completely inhibited all the effects. In summary, nanoparticles can be used as transporters for fleeting lipids, such as SPMs, and therefore expand the array of possible therapeutic agents. Thus, the Fpr2–Cd206–TAM receptor axis may be a suitable target for strengthening the capillary barrier, removing endoneurial fibrinogen and boosting pain resolution in patients with chronic neuropathic pain.

**Supplementary Information:**

The online version contains supplementary material available at 10.1007/s00401-024-02840-9.

## Introduction

Traumatic peripheral nerve injury elicits neuropathic pain followed by regeneration, with axonal regrowth, remyelination and target organ innervation. While many patients recuperate, some fail to recover and suffer long-lasting chronic pain. In the last few decades, research has provided valuable insights into chronification mechanisms, but healing and resolution of pain are less well understood [12]. Regeneration failure includes incomplete restoration or miswiring of sensory and nociceptive fibres, causing neuropathic pain [21].

The initial pathology after chronic constriction injury (CCI) includes oedema, Wallerian degeneration and disruption of the perineurium, with macrophage infiltration and endothelial proliferation observed within the first week. The long-term effects involve significant loss of nerve fibres, axonal sprouting and fibrous tissue formation around the ligatures [60]. Vascular changes are evident as endothelial cell proliferation and vessel narrowing in a distinct time course: distended vessels 1 day after surgery, followed by progressive thickening of vessel walls and reduced luminal size from day 7 [59].

Immune cells are believed to play an important role in the switch to pain resolution which can occur independently of the recovery of tissue architecture and function [60]. While sensory or motor defects persist, the regeneration phase can emerge with mild or no pain. Immune cells control the pro-resolution switch, including macrophages and T cells, releasing anti-inflammatory cytokines [20, 30, 58], specific subtypes of spinal microglia [17] and mitochondria or extracellular vesicles from anti-inflammatory macrophages [69]. These highly regulated processes balance the switch for chronification or resolution [44]. However, the temporal course in nerve tissues is not thoroughly understood.

Nerve barriers are composed of perineurial, endothelial and Schwann cells that shield the delicate nerve milieu from external stimuli [40, 42, 52]. Under homeostatic conditions, nerve barriers ensure proper nerve function and conduction through tight junction proteins, such as claudins, pericytes and endoneurial macrophages [37, 52]. Endoneurial oedema represents one of the earliest signs of nerve damage and barrier leakage in patients with inflammatory and non-inflammatory polyneuropathy and preclinical models [60, 67], which manifests as endoneurial deposition of plasma proteins, such as fibrinogen [56], and loss of myelin barrier proteins [10].

After nerve injury, barrier breakdown—evident as tight junction protein loss and immune cell infiltration—facilitates the proper removal of myelin debris. Following successful clearance, nerve barriers need to be resealed to protect the regenerated sensitive structures of the nerve milieu. The proposed factors that promote barrier resealing are TAM receptors (Tyro3, Axl and Mer), which are part of a group of receptor tyrosine kinases that control efferocytosis in both macrophages and Schwann cells [5, 19]. TAM receptors mediate the resolution of inflammation and promote the production and synthesis of anti-inflammatory and specialized pro-resolving mediators [68]. These SPMs—other factors for barrier resealing—derived from omega-3 and omega-6 polyunsaturated fatty acids, actively resolve inflammation [63]. SPMs such as maresins, protectins and resolvins activate seven different cell surface G-protein coupled receptors (GPCRs), including formyl peptide receptor 2 (FPR2) and G protein-coupled receptor 37-like 1 (GPR37L1), thereby ameliorating inflammatory and neuropathic pain [4, 62], endothelial cell activation and vascular smooth muscle cell remodelling [15].

In this study, we explored the interplay between the nervous system, barrier function and the immune system to create a temporal signature of pain resolution after traumatic nerve injury. Specifically, we employed in-depth lipidomics, barrier mapping and pathway analysis and therapeutically used SPM-laden nanoparticles to foster pain resolution.

## Materials and methods

### Animal model

All animal experiments were approved by the Government of Lower Franconia, Germany (55.2.2-2532-2-612–06.04.2018 and 55.2.2-2532-2-1547–25.04.2022, Regierung von Unterfranken). Wistar rats (Janvier Labs, Le Genest-Saint-Isle, France) were maintained under pathogen-free conditions with a controlled light cycle, temperature and humidity (14:10 h light/dark cycle, 20–24 °C, 45–65% humidity). Rats (200–250 g) were provided standard chow and water ad libitum and were randomly assigned to the experimental groups. For chronic constriction injury (CCI), the rats were deeply anaesthetized with 2–4% isoflurane. The right sciatic nerve was surgically exposed, and four loose silk ligatures were placed around it with a spacing of approximately 1 mm. The incision was closed using sutures. Animals with auto-mutilation were excluded from the experiments. Sham-operated rats served as controls. All experiments were performed in accordance with ethical regulations.

### Perineurial injection

Perineurial injections were performed under isoflurane anaesthesia. A blunt cannula attached to a nerve stimulator was inserted through the skin after prepuncture with a 22-Gauge cannula. When proximity to the nerve was confirmed by foot twitching, 0.3 ml of 500 nmol BML-111/phosphate-buffered saline (PBS; Merck KGaA, Darmstadt, Germany, cat. no. SML0215-25MG) or 1 mg RU-301/64% DMSO/PBS (Biozol, cat. no. MCE-HY-119039) or vehicle was injected. For the injection of nanoparticles, the volume was 0.1 ml.

### Behavioural testing, reflexive and non-reflexive

The von Frey test was performed to assess mechanical allodynia using the Dixon’s up-and-down method. Von Frey filaments (Aesthesio® set, Ugo Basile SRL, Gemonio, Italy) were applied to the plantar surface of each hind paw for 1–3 s so that the filaments were bent to a 45° angle. Single paws were tested with a recovery time of at least 30 s between applications. For analysis, the means of the filament forces with positive reactions were averaged per paw.

Thermal hypersensitivity was assessed by the Hargreaves test. The light source of a Plantar Analgesia Meter (Model 400 Heated Base from IITC Life Science, Woodland Hills, California, USA) was applied to the plantar surface of each hind paw with a cut-off latency of 20 s. Withdrawal latencies were recorded twice (at least 30 s intervals) and averaged for analysis.

Motor activity was assessed by voluntary wheel running (Model BIO-ACTIVW; Bioseb, Vitrolles, France). Once per week, the rats were individually placed in cages with voluntary running wheels for 24 h. The activity parameters, including distance, speed and acceleration, were recorded using the BIO-ACTIVW-SOFT software (Bioseb).

The CatWalk XT system (Noldus, Wageningen, the Netherlands) was used for gait analysis. The rats were allowed to move freely on the CatWalk until three valid runs were recorded with a camera. Runs with a run duration of 0.5–5 s and a maximal run speed variation of 60% were classified as valid. The ratios of footprint area and standing time between the right and left hind paws were calculated. Data from three valid runs were averaged for the analysis.

### Reverse transcription-quantitative PCR

To isolate RNA from the snap-frozen sciatic nerve tissue (the site of ligation in CCI), we used the RNeasy Micro Kit (#74,004, Qiagen, Venlo, the Netherlands) according to the manufacturer’s instructions. RNA concentration was determined using a NanoDrop ND 2000 spectrophotometer (Thermo Fisher Scientific). 1000 ng (for the 1–6-week characterization) and 500 ng of total RNA were reverse-transcribed to cDNA using the High-Capacity cDNA Reverse Transcription Kit, according to the manufacturer’s instructions (Thermo Fisher Scientific). For quantitative polymerase chain reaction (qPCR), the following TaqMan gene expression assays with FAM-labelled probes were used (Applied Biosystems, Thermo Fisher Scientific): *Gapdh* (Rn01462662_g1), *Tnfα* (Rn99999017_m1), *Cd68* (Rn01495634_g1), *Plat* (Rn01482578_m1), *Tjp1* (Rn02116071_s1), *Cldn1* (Rn00581740_m1), *Cldn5* (Rn01753146_s1), *Cldn12* (Rn04219013_m1), *Cldn19* (Rn01416537_m1), *Mpz* (Rn00566746_m1), *Gpr18* (Rn01493247_m1), *Gpr37* (Rn00589441_m1), *Gpr37l1* (Rn00595762_m1), *CmklR1/ChemR23* (Rn00573616_s1)*, Fpr2* (Rn03037051_gH), *Lgr6* (Rn01490727_m1), *Arg1* (Rn00691090_m1), *Mrc-1/Cd206* (Rn01487342_m1) and *Nlrp3* (Rn04244620_m1). qPCRs were run on a StepOnePlus real-time PCR system (Applied Biosystems) using the following program: 40 cycles at 95 °C for 1 s and 60 °C for 20 s. The samples were run in triplicate and averaged for the analysis. mRNA abundance relative to the reference gene *Gapdh* was presented as 2^−ΔCt^.

### Permeability assessment: perineurial barrier, myelin barrier and endoneurial vessels

To assess perineurial permeability, endings of the sciatic nerve were sealed with Vaseline and immersed in 1 ml of 5% Evan’s Blue albumin (EBA; 5% bovine serum albumin, 1% Evans Blue) and 3% sodium fluorescein (NaFlu) in PBS for 15 min, followed by 5% EBA for 45 min at room temperature (RT). After washing with PBS, the nerves were fixed in 4% paraformaldehyde (PFA) for 1–3 h, cryoprotected in 30% sucrose at 4°C overnight and frozen in Tissue-Tek® O.C.T.™ Compound (Sakura, Alphen aan den Rijn, the Netherlands). Ten-micrometre sections were cut using a cryostat (Leica CM3050S; Leica Biosystems, Wetzlar, Germany). Immunofluorescence images were acquired using a microscope (BZ-9000, Keyence Deutschland GmbH, Neu-Isenburg, Germany), and the intensity was measured.

For myelin barrier assessment, the epi-perineurium was removed from the nerves proximal from the ligation, which were sealed with Vaseline at their endings [9]. After incubation in artificial cerebrospinal fluid (10 mM HEPES, 110 mM NaCl, 17.8 mM NaHCO_3_, 4 mM MgSO_4_, 3.9 mM KCl, 3 mM KH_2_PO_4_, 1.2 mM CaCl_2_ and 10 mM dextrose) containing 0.5% 70 kDa fluorescein isothiocyanate-dextran (FITC-Dextran; Sigma-Aldrich, St. Louis, MO, USA) for 1 h at 37 °C, the nerves were washed, fixed in 4% PFA for 5 min at RT and teased to single nerve fibres on microscopy slides. Until mounting with Aqua-Poly/Mount medium, the slides were stored at -20 °C. Fluorescence images were then acquired for the analysis.

To assess the permeability of the endoneurial blood vessels, rats received an intravenous injection of 1 ml 5% EBA per 100 g body weight [43]. After 30 min, the proximal and distal parts of the sciatic nerve were snap-frozen in Tissue-Tek® O.C.T.™ Compound. Immunofluorescence images of 10-µm sections were acquired, and the endoneurial intensity was measured.

### In vitro assessment of barrier recovery by resolvin D1 and permeability for fibrinogen

Primary human dermal microvascular cells (HDMECs; PromoCell, Heidelberg, Germany) were employed as endothelial/endoneurial model and seeded on Millipore filter inserts (Merck-Millipore, PCF-filters, pore size 0.45 µm, area 0.6 cm^2^) and grown until confluent and stable in transendothelial resistance (TER). The cells were then challenged with 500 U/ml TNF-α (Peprotech) for 24 h in parallel to untreated controls. Then, TNF-α concentration was reduced to avoid the induction of apoptosis to 100 U/ml, and some filters were additionally incubated with 500 nM RvD1 (Cayman Chemicals/Biomol GmbH, Hamburg, Germany) for a further 24 h. Permeability for fibrinogen was determined, using FITC-labelled fibrinogen (LOXO GmbH, Dossenheim, Germany), which was added at a concentration of 0.58 µM apically to the filters. After 4, 5 or 6 h, concentrations of FITC-fibrinogen in the basal compartments were measured fluorometrically (Tecan Infinite M200, Tecan, Switzerland). To analyse potential changes in the transcellular passage, e.g. by endocytosis, the cells were harvested from the filters after washing with PBS and were dissolved using total lysate buffer (10 mM Tric-Cl pH 7.5, 150 mM NaCl, 0.5% Triton X-100, 0.1% SDS, protease inhibitors). The intracellular concentration of FITC-fibrinogen was detected at the Tecan photometer. Tracer fluxes and apparent permeabilities were calculated from the concentrations of FITC-fibrinogen.

### Liquid chromatography-tandem mass spectrometry

The SPMs were determined as described in a previous study by *Toewe *et al*.* [65] after some modifications of the extraction method. After dissection, sciatic nerve tissues were snap-frozen in liquid nitrogen. The tissue samples were homogenized in water:ethanol (75:25, v/v) to a tissue homogenate of 0.1 mg/μl using a swing mill (Retsch, Haan, Germany) with five zirconium oxide grinding balls (25 Hz for 2.5 min). Three hundred microlitres of the tissue homogenates (in total 30 mg tissue) was spiked with the internal standard solution and extracted using solid-phase extraction with Express ABN cartridges and Extrahera (both from Biotage). Afterwards, the sample extracts were measured using a liquid chromatography-tandem mass spectrometry (LC-MSMS) system comprising an Agilent 1200 LC system and a 5500 QTRAP mass spectrometer (Sciex). Chiral chromatographic separation was achieved using a Lux Amylose-1 column (250 × 4.6 mm, 3 µm, Phenomenex) with water:FA (99.9:0.1, v/v) as solvent A and ACN:MeOH:FA (95:4.9:0.1, v/v/v) as solvent B in gradient elution mode. Data acquisition and evaluation were performed using Analyst 1.6.2 and MultiQuant 3.0, respectively (both from Sciex).

### Production of nanoparticles

Nanoparticles were produced as previously described [50]. Briefly, PLGA-PEG-biotin (Nanosoft Polymers; PLGA molecular weight = 10 kDa, PEG molecular weight = 2 kDa) was dissolved in dimethylformamide (100 mg/ml), and 3.76 μg of RvD1 (Cayman Chemical, Ann Arbor, USA; dissolved in ethanol at 0.1 mg/ml) was added to the polymer solution. Next, 500 µL of this polymer–resolvin mixture was added dropwise to 10 mL of PBS. Unloaded control nanoparticles were prepared by dropwise addition of a polymer solution (without resolvin) to PBS. The nanoparticles were stirred for 4–5 h, concentrated by centrifugation using Amicon Ultra-15 centrifugal filter units and filtered through sterile 0.45-μm syringe filters.

### Dialysis of nanoparticles to measure resolvin D1 release

Slide-A-Lyzer™ mini dialysis unit 20 K MWCO (Thermo Fisher Scientific, Darmstadt, Germany; ref: 69,590) was loaded with 100 µL of RvD1-laden nanoparticles. The devices were placed in 1.1 mL of PBS. The prepared tubes were stirred in a thermomixer at 300 rpm and 37 °C for 1, 3 and 6 h or 1, 2 and 3 days. The dialysates were stored at -80 °C until shipment for analysis by LC-MSMS.

### Immunofluorescence of rat nerve tissues

The nerves were snap-frozen in Tissue-Tek® O.C.T.™ Compound. The 10-µm sections were fixed in a -20 °C acetone bath. Blocking and permeabilization were performed using PBS containing 0.3% Triton X-100, 0.1% Tween20 and 10% donkey serum for 1 h at RT. The sections were incubated with goat anti-fibrinogen (1:100; antibodies-online GmbH, Aachen, Germany, cat. no. ABIN458743) primary antibodies overnight at 4 °C. Suitable secondary antibodies were applied for 1 h at RT: donkey anti-goat IgG Alexa Fluor 594 (1:600; cat. no. A32758, Thermo Fisher Scientific). Before mounting with Aqua-Poly/Mount medium (Vector Labs, Burlingame, CA, USA), nuclei were stained with DAPI. In this study, we used a polyclonal fibrinogen antibody that binds to various fibrin(ogen) (degradation) products. In the following section, we will use the term fibrinogen for fibrin.

### Human nerve tissues

The human sural nerves were obtained for diagnostic purposes. Nerve material that was left after diagnostic workup was used for the study with approval from the Ethical Committee of the University of Würzburg (238/17). For patient details, please refer to Supplementary Table ST1. Sixteen biopsies from patients with neuropathies were obtained for immunofluorescence, freshly frozen in OCT and stored at  – 80 °C. The 10-µm sections were cut. For staining, the sections were thawed, dried and fixed in 4% PFA for 10 min at RT. After washing, the sections were blocked with 10% donkey serum, 0.1% TritonX-100 and 0.3% Tween20 in PBS for 1 h at RT. Rabbit anti-tissue plasminogen activator (tPA; 1:100; 10,147–1-AP, Proteintech) and sheep anti-fibrinogen (1:200; ab118533, Abcam) antibodies were incubated overnight at 4 °C. Suitable secondary antibodies (1:600; anti-rabbit Alexa Fluor 488, Invitrogen, #A32731; anti-sheep Alexa Fluor 647, Invitrogen, #A21448) were incubated for 1 h at room temperature. Before mounting with Aqua-Poly/Mount medium (Vector Labs), the nuclei were stained with Hoechst33342.

RNAseq datasets from human intact and transected sural nerve fibres obtained from the participants of a nerve transplantation clinical trial for Parkinson’s disease were reported by Welleford et al. [72] and made publicly available via the UK knowledge website (https://uknowledge.uky.edu).

### Image analysis

Nerves from the permeability assays and fibrinogen staining were imaged using a fluorescence microscope (BZ-9000; Keyence Deutschland GmbH, Neu-Isenburg, Germany). The images were further analysed using ImageJ software [57]. The endoneurial region of each image was manually selected, and the mean intensity was measured. For each sample, three images were analysed and averaged.

For the teased fibres, brightfield images were acquired to place a 5-µm-diameter circle as the region of interest (ROI) in the internodal region of the fibres and three ROIs for background subtraction. These ROIs were transferred to their corresponding fluorescence images, and the mean intensities of the ROIs were measured. The intensity of the background ROIs was averaged and subtracted from each internodal ROI.

Image acquisition for human nerve tissues was performed using an Axio Imager.M2 fluorescence microscope coupled with an Axiocam 506 mono camera (Zeiss, Oberkochen, Germany). For each sample, 2–3 images were analysed and averaged.

### Statistical analysis

Statistical analyses were performed using GraphPad Prism version 9.3.0 Windows (GraphPad Software, San Diego, CA, USA). For animal behaviour tests, two-way repeated-measures ANOVA with Tukey’s multiple comparison test was performed. The tests performed included Mann–Whitney U test as a non-parametric test or Student’s t test (incl. Welch’s correction for unequal variance) and two-way ANOVA and post-hoc test for multiple groups (see figure legends). Statistical significance was set at p < 0.05. All data were expressed as mean ± SEM.

## Results

### Resolution of thermal and mechanical hyperalgesia in chronic constriction injury from 4 to 6 weeks

**Fig. 1:**
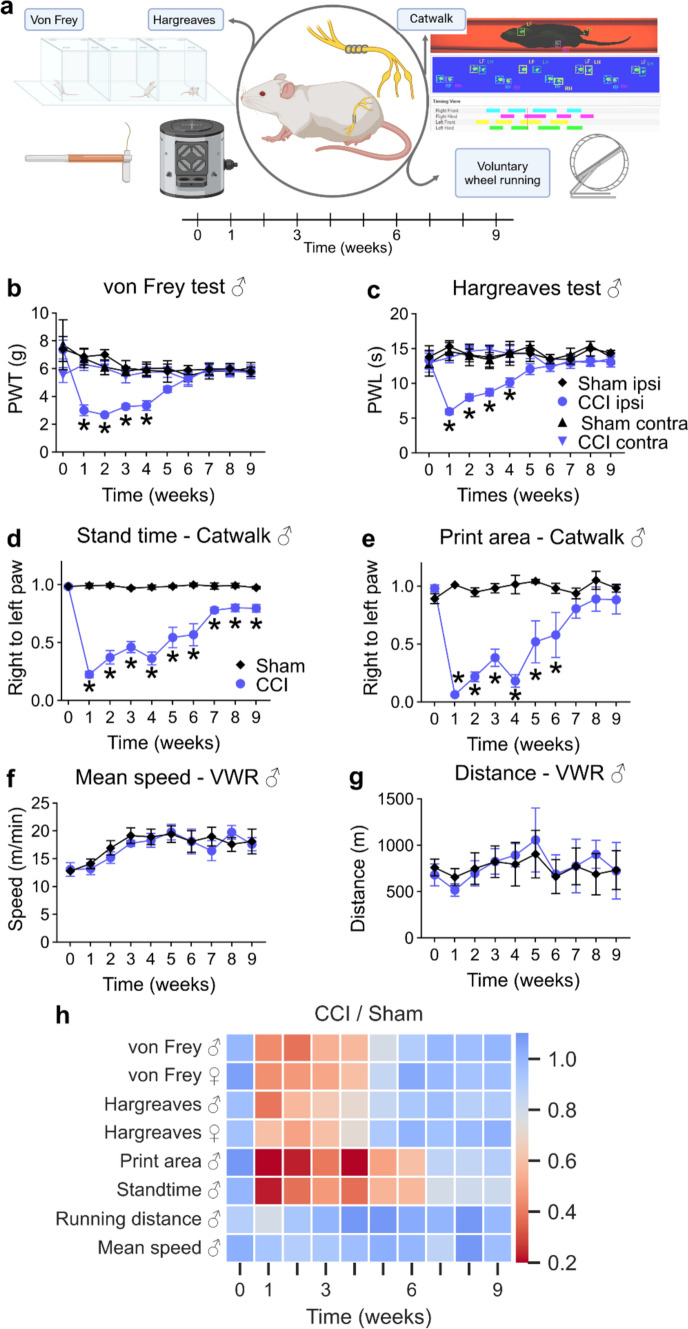
Neuropathic pain resolves 6 weeks after nerve injury. **a** Schematic illustration of the experimental setup: Male and female Wistar rats underwent CCI or sham surgery and were examined using the displayed tests throughout 9 weeks. **b** Mechanical allodynia and **c** thermal hypersensitivity were assessed weekly (n = 6–8). **d** The print area and **e** stand time presented as the ratios of values from right to left hindpaws were recorded using the Catwalk gait analysis system (*n* = 6–7). **f** The covered distance and **g** the mean speed of CCI and sham animals were recorded with voluntary running wheels (*n* = 8). **h** The summary of all behavioural tests including von Frey and Hargreaves from both male and female rats. The heatmap shows the ratios between the arithmetic means of CCI and sham groups. Data in graphs are shown as mean ± SEM, *p < 0.05. Two-way repeated-measures ANOVA with Tukey’s multiple comparison. *PWT* paw withdrawal threshold, *PWL* paw withdrawal latency, *CCI* chronic constriction injury, *VWR* voluntary wheel running, *ANOVA* analysis of variance

We characterized the pain course using reflexive and non-reflexive tests over 9 weeks to determine the period of resolution of neuropathic pain after CCI in Wistar rats (Fig. 1a). Mechanical and thermal thresholds decreased until week 4 after CCI compared with sham animals and completely recovered at week 6 in both males (Fig. 1b, c) and females (SFig. 1). Assessment of the mean print area and the stand time in gait analysis can serve as a measure of pain [24]. The gait pattern drastically changed after CCI, while the gait of the sham-operated rats remained unaffected (Fig. 1d, e). Gait recovery took a similar but delayed course compared with reflexive tests. The stand time did not completely return to baseline levels after 9 weeks, which is thought to result from nerve damage rather than pain. Despite their impaired gait, CCI animals demonstrated normal physical activity throughout the entire time course, using a voluntary running wheel (Fig. 1f, g).

Based on the curves for mechanical and thermal hyperalgesia and footprint analysis, pain resolution started from week 3 to week 4 after CCI, whereas the end of the resolution was evident at week 6 (Fig. 1h). Thus, we defined week 1 after surgery as maximal hypersensitivity, week 3 as the flexion point of resolution, week 6 as the end of pain resolution and week 9 as the nerve regeneration phase.

### Capillaries reseal for fibrinogen in parallel to pain resolution

**Fig. 2:**
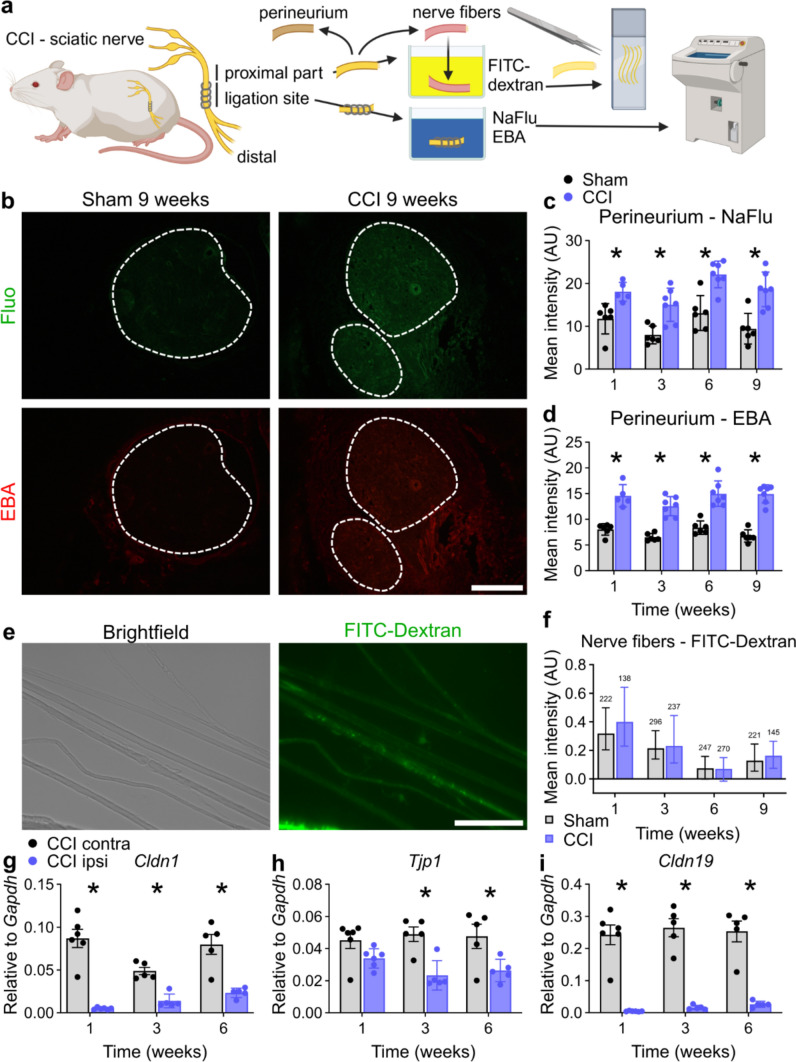
The perineurial barrier remains open up to 9 weeks, while the myelin barrier stays intact. **a** Depiction of two ex vivo immersion techniques for analysing the perineurial and myelin barriers after CCI or sham. **b** Representative images of sciatic nerve cross sections and **c, d** quantification of endoneurial fluorescence intensity of nerves immersed in Fluo (332 Da) and Evans blue albumin (EBA, 68 kDA) ex vivo. Dashed lines indicate the endoneurial regions. Scale bar: 300 µm. **e** Representative brightfield and fluorescence images of teased nerve fibres from the proximal part of the sciatic nerve after immersion in 70 kDa FITC-dextran. Scale bar: 100 µm. **f** Quantification of the fluorescence intensity measured in the internodal regions of the teased fibres. (*n* = 138–296 from 5 to 6 animals per group, pairwise Mann–Whitney U tests). The relative gene expression of **g** *Cldn1*, **h** *Tjp1* and **i** *Cldn19* in the ligation parts of ipsilateral and contralateral sciatic nerves after 1, 3 and 6 weeks after CCI (n = 5–6). All data are shown as mean ± SEM, *p < 0.05 compared to control at the indicated time points, two-way ANOVA with Šidák’s multiple comparisons unless indicated otherwise

To determine whether pain resolution coincided with the resealing of any of the nerve barriers, we performed a battery of permeability tests and tight junction protein analysis (Fig. 2a).

Hyperpermeability of the perineurium persisted for Fluo (332 Da) and EBA (69 kDa) for up to 9 weeks after CCI (Fig. 2b–d). To assess the state of the myelin barrier in the proximal, non-degenerating part of the nerve, we immersed desheathed nerve fibres in FITC-dextran (70 kDa). Dye leakage through the myelin barrier was similar after nerve injury compared with that in the sham group (Fig. 2e, f). Perineurium breakdown was reflected by low levels of *Cldn1* and *Tjp1* (Fig. 2g, h) in the ligated region, whereas *Cldn19* expression was heavily reduced (Fig. 2i) in accordance with *Mpz* levels (SFig. 2b).

**Fig. 3:**
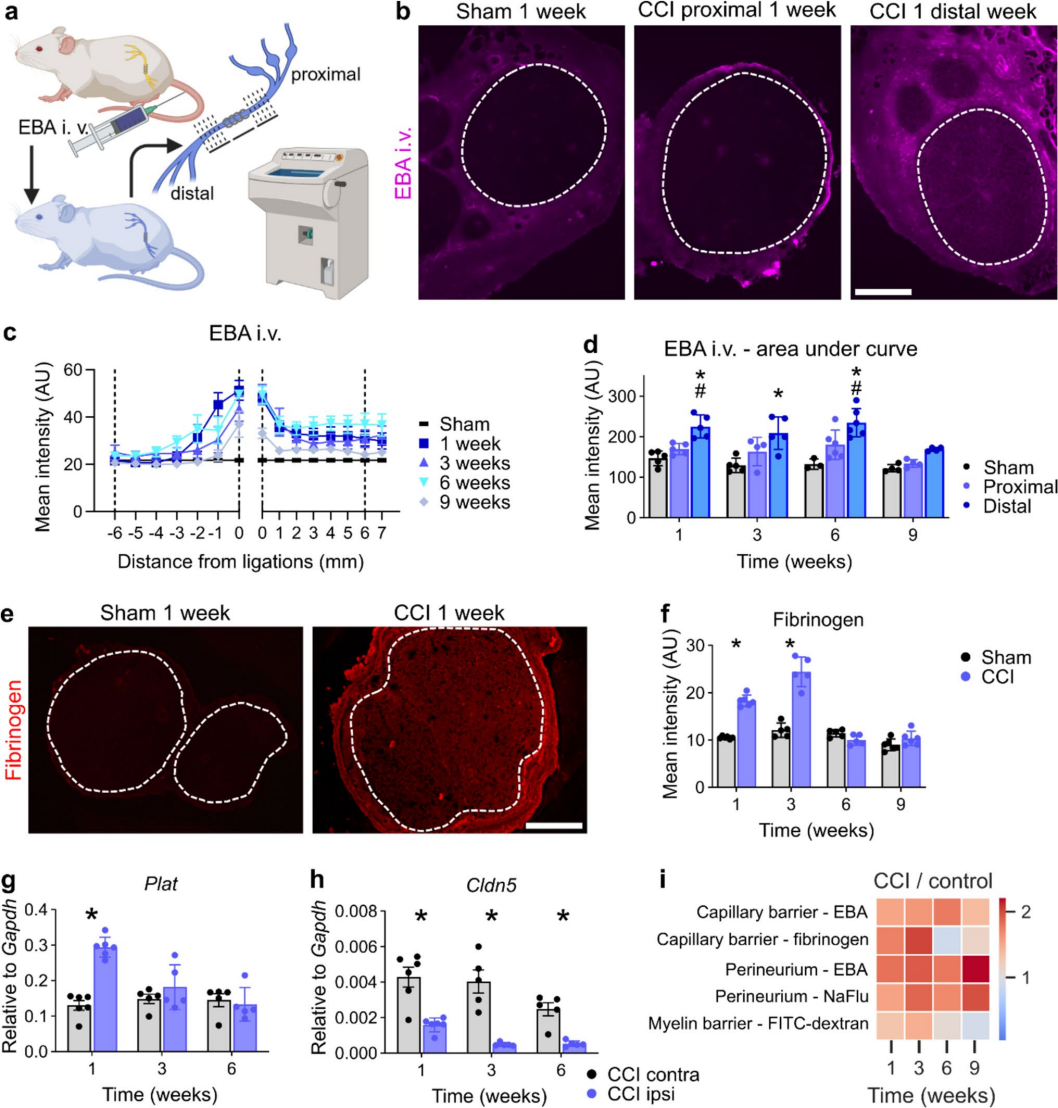
The capillary barrier reseals specifically for fibrinogen in parallel with neuropathic pain. **a** Schematic illustration of the capillary permeability test: Evans blue albumin (EBA, 68 kDa) was intravenously injected. Cross sections at distinct positions distal and proximal from the ligations were collected and analysed. **b** Representative images of sciatic nerve cross sections. The dashed lines indicate the endoneurial regions. Scale bar: 300 µm. **c** Depiction of the spatial and temporal EBA extravasation into the endoneurium through endoneurial vessels. Sham values were averaged and plotted on each position as reference. Dashed lines mark the positions that were used for the calculations for the areas under the curve in **d** Quantification analysis of the areas under the curves distal and proximal from 0 to 6 mm from the ligations were plotted (*n* = 3–6). *distal vs. sham; #distal vs. proximal *p* < 0.05 two-way ANOVA with Bonferroni’s multiple comparison test. **e** Representative images of fibrinogen immunoreactivity in the sciatic nerve 1 week after surgery. The dashed lines indicate the endoneurial region. **f** Quantification of the intensity of fibrinogen immunoreactivity within the endoneurial regions (*n* = 5–6). Scale bar: 300 µm. Relative gene expression of **g** *Plat* and **h** *Cldn5* in sciatic nerves after CCI (*n* = 5–6). **i** Summary of all permeability tests. The heatmap displays the ratios between the arithmetic means of CCI and sham groups. All data are shown as mean ± SEM, *p < 0.05 two-way ANOVA with Šidák’s multiple comparisons

The integrity of the intraneural blood vessel barrier was compromised from weeks 1–6 for EBA and reestablished at week 9 (Fig. 3a–d). Intravenously injected EBA leaked through the blood vessels at the ligation and distal parts, whereas the blood–nerve barrier (BNB) in the proximal parts further away from the ligations was unaffected. Accordingly, *Cldn5* was reduced in the ligated region (Fig. 3h); exemplary fluorescence images of Cldn5 are depicted in SFig. 3. *Cldn12*, another tight junction protein, was also reduced and did not recover with pain resolution (SFig. 2a).

Endoneurial leakage of blood proteins is a well-known hallmark of polyneuropathy [56]. Fibrinogen (340 kDa) is a blood coagulation factor, produced in the liver, which polymerizes to fibrin upon tissue damage. tPA (gene: *Plat*) activates plasminogen to degrade fibrin. In injured nerves, fibrinogen accumulated endoneurially and *Plat* was upregulated until 3 weeks (Fig. 3e–g). When hypersensitivity was resolved, both fibrinogen and *Plat* returned to sham levels, indicating complete recovery of the BNB in terms of fibrinogen leakage. The capillary barrier was impermeable to fibrinogen at the time of complete pain resolution.

To detect endoneurial fibrinogen, we used an antibody binding to various fibrin(ogen) (degradation) products. Endoneurial fibrinogen correlated with the pain course in the analysis of the barrier portfolio (Fig. 3i), indicating that the presence of fibrinogen, a pro-inflammatory and pro-nociceptive molecule [1, 31] that penetrates the capillary of the BNB into the endoneurium, might explain the pathophysiology of the switch to pain resolution.

### Human nerves contained more endoneurial fibrinogen in painful neuropathy

**Fig. 4:**
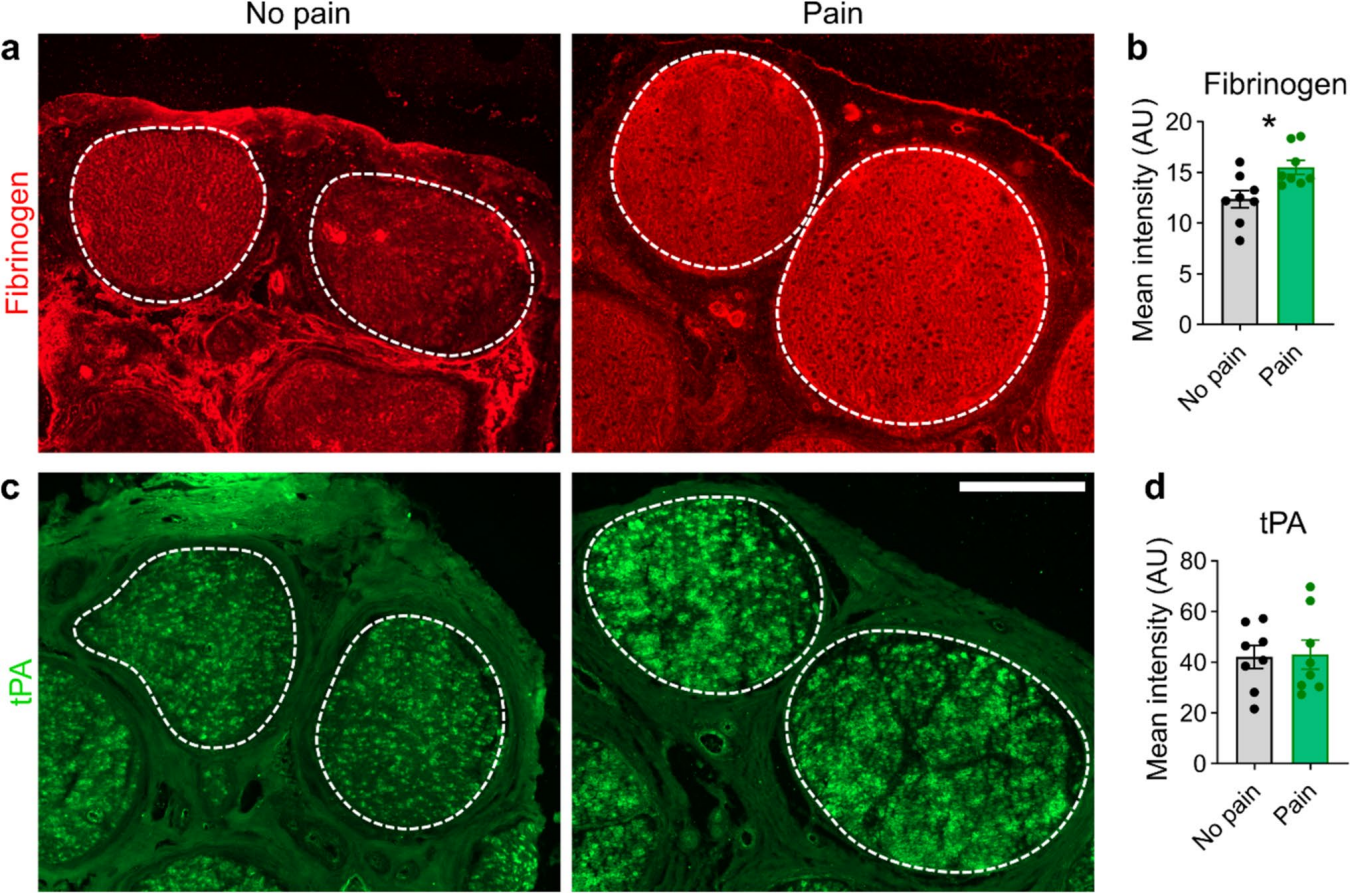
More endoneurial fibrinogen in sural nerve biopsies from patients with painful polyneuropathies. Sural nerve biopsies from patients were immunofluorescently stained. Representative images of human sural nerves stained for **a** fibrinogen and **c** tissue plasminogen activator (tPA). The dashed lines indicate the endoneurial regions. Scale bar: 300 µm. **b, d** Quantifications of the signal intensity of fibrinogen and tPA. Patients were grouped by pain occurrence (*n* = 16, Student’s t tests with Welch’s correction)

To validate our preclinical findings in a translational approach, we analysed sural nerve biopsies of patients with different polyneuropathies (Table 1). Patients were aged 69 ± 7.8 years and were mainly male (10 males and 6 females), with a disease duration of 52 ± 44.1 months and a clinical severity of neuropathy of 2 ± 1.2 (Overall Disability Sum Score [ODSS] score, 0 = no sign of disability to 12 = most severe disability). The demyelinating and axonal neuropathies were almost evenly distributed. Half of the patients (8 of 16) had pain at the time of nerve biopsy, while the other half did not. Follow-up information was available from only six patients. Three of them had pain, of which only one was painless several years later. We immunostained the nerves and compared the fibrinogen and tPA signal intensities (Fig. 4a, c). Patients with painful polyneuropathy showed higher fibrinogen immunoreactivity in the endoneurium, whereas the tPA levels did not differ (Fig. 4b, d). These results suggest that fibrinogen may also play a role in the persistence of pain in human patients with painful neuropathy with longer duration and lack of clearance.

**Table 1:** Patient demographic and clinical characteristics of painful and non-painful neuropathies

Age	Sex	Diagnosis	Duration (months)	ODSS	Pain	Histology
77	F	CIAP	48	2	Yes	Mixed
72	M	CIDP/atypical	12	2	Yes	Demyelinating
71	M	CIAP	48	2	Yes	Axonal
67	F	Idiopathic PNP	24	3	Yes	Axonal
73	M	CIDP/atypical	36	6	yes	Axonal
57	F	Idiopathic PNP	6	1	Yes	Axonal
73	M	CIDP/atypical	9	3	Yes	Demyelinating
62	F	Idiopathic PNP	66	1	Yes	Axonal
64	M	CMT	36	2	No	Axonal
59	F	CIDP	120	3	No	Demyelinating
71	M	Idiopathic PNP	120	2	No	Demyelinating
66	F	Idiopathic PNP	120	2	No	Demyelinating
76	M	CIDP	12	2	No	Demyelinating
53	M	CMT	48	3	No	Demyelinating
80	M	CIDP/atypical	7	2	No	Axonal
76	M	CIAP	120	1	No	Axonal

### Lipidomics: 15R-HETE culminates selectively at the switch for pain resolution

**Fig. 5:**
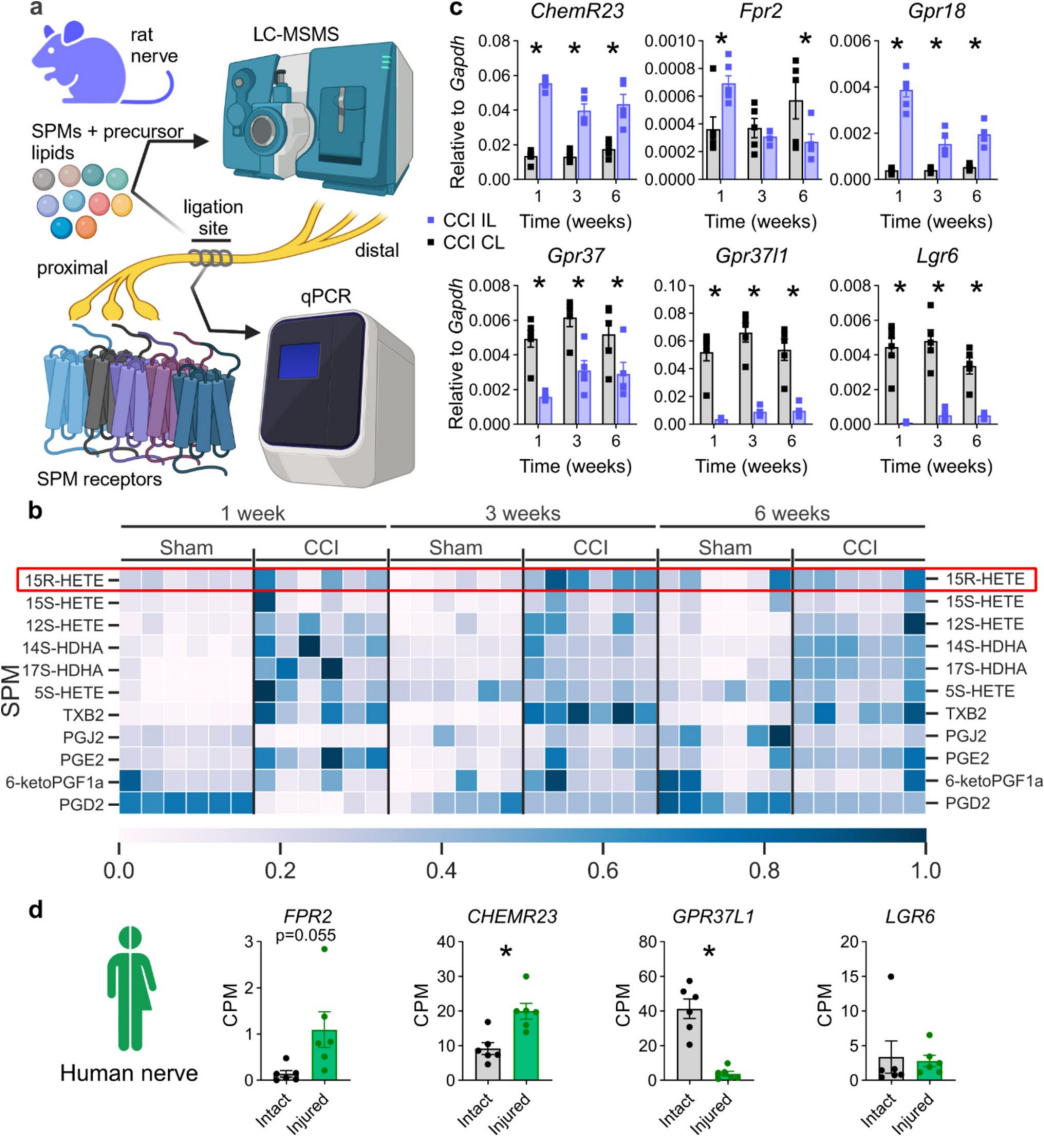
Unbiased lipidomic analysis selectively identified higher levels of the LXA4 precursor 15R-HETE at 3 weeks, initiating the resolution phase. **a** Arachidonic-, docosahexaenoic- and eicosapentaenoic acids-derived metabolites were measured by LC-MSMS in the sciatic nerve, and their cognate receptors were assessed using qPCR. **b** Relative levels of the 11 detected lipid metabolites are depicted. Only 15R-HETE (circled in red) had a significant upregulation in CCI specifically at 3 weeks compared with sham. **c** Relative mRNA expression of the respective SPM receptor after CCI; *CL* contralateral, *IL* ipsilateral. All data are shown as mean ± SEM, *n* = 5–6, two-way ANOVA with Šidák’s multiple comparisons. *p < 0.05; ANOVA: analysis of variance. **d** Expression levels as counts per million (CPM) of the SPM receptors from intact and injured human sural nerve tissues collected 2 weeks after a full transection injury (*n* = 6, Student’s *t* tests with Welch’s correction), as obtained from previously published bulk RNAseq data [72]

We hypothesized that pain resolution is initiated by resealing of the nerve barriers which in turn might be induced by SPMs [35]. To identify the lipids driving the lipid mediator class switch from pro-inflammatory to pro-resolving and their respective receptors, we performed a lipidomic analysis by LC-MSMS and qPCR of the sciatic nerve (Fig. 5a). We analysed 34 lipids derived from arachidonic acid (AA), docosahexaenoic acid (DHA) and eicosapentaenoic acid (EPA), and detected 11 of them. Although pro-inflammatory prostaglandin E2 (PGE2) and thromboxane B2 (TXB2) were more abundant after injury, prostaglandin D2 (PGD2) levels were reduced. Among the three hydroxyeicosatetraenoic acid (HETE) precursor lipoxins (5S-, 15S- and 15R-HETE), only 15R-HETE selectively increased at the beginning of pain resolution (Fig. 5b). Specialized pro-resolving mediators, such as neuroprotection D1 (NPD1), resolvin D1 (RvD1), resolving D2 (RvD2) and maresin 1 (MaR1), could not be detected. Other lipids from pro-resolving synthesis pathways, such as 17S-HDHA (a precursor of RvD1 and 2) and 14S-HDHA (derived from DHA), showed higher concentrations, but not selectively, at 3 weeks (Fig. 5b). Lipids derived from EPA (18R-HEPE) were not detected.

To further evaluate the potential of the aspirin-triggered lipoxin A4 (AT-LXA4) precursor 15R-HETE to resolve pain, we determined the mRNA expression of its receptor, formyl peptide receptor 2 (*Fpr2*), and other known SPM receptors *Gpr18, Chem23r, Gpr37* and *Lgr6* (Fig. 5c)*. Gpr18* and *ChemR23* were upregulated, whereas *Lgr6*, *Gpr37* and *Gpr37l1* were downregulated in the rat sciatic nerve. *Fpr2* was not abundant but was increased at 1 week and decreased 6 weeks after injury (Fig. 5c), but it is known to be expressed in rat macrophages [53]. To assess the translatability of our findings, we reanalysed data from bulk RNA sequencing (RNAseq) experiments from human transected nerves compared to naive human nerves [72]. In human nerves, *FPR2* and *CHEMR23* were increased, *GPR37L1* was downregulated, while *LGR6* was unchanged after injury (Fig. 5d). To further elucidate which cell type expresses the different receptors, we analysed receptor expression in purified in vitro cultured Schwann cells, the most abundant endoneurial cell type. *FPR2/Fpr2* was not expressed in human or rat Schwann cells before and after differentiation (SFig. 4) [6]. All other receptors (*CHEMR23*, *GPR18*, *LGR6, GPR37* and *GPR37L1*) were expressed in Schwann cells of human and/or rat origin as shown in SFig. 4. Since *Fpr2* was absent in Schwann cells, we next explored the cellular source of *Fpr2* after CCI. To this end, we reanalysed a previously published single-cell RNAseq (scRNAseq) dataset [33] employing publicly available experimental results on gene expression at different time points after CCI (i.e. naive nerves as well as nerves 3, 12 and 60 days after CCI; SFig. 5a). In our reanalysis, we were able to identify the types of major cell populations, such as macrophages, Schwann cells and epineurial fibroblasts (SFig. 5b, c). Similar to our previous observations (Fig. 5c), *Fpr2* abundance was in general very low (SFig. 5d) but was significantly enriched in macrophages when compared to other cell types (SFig. 5e), indicating that the activation of Fpr2 primarily occurs in macrophages.

### Fpr2 activation by BML-111 accelerates pain resolution and the degradation of fibrinogen

**Fig. 6:**
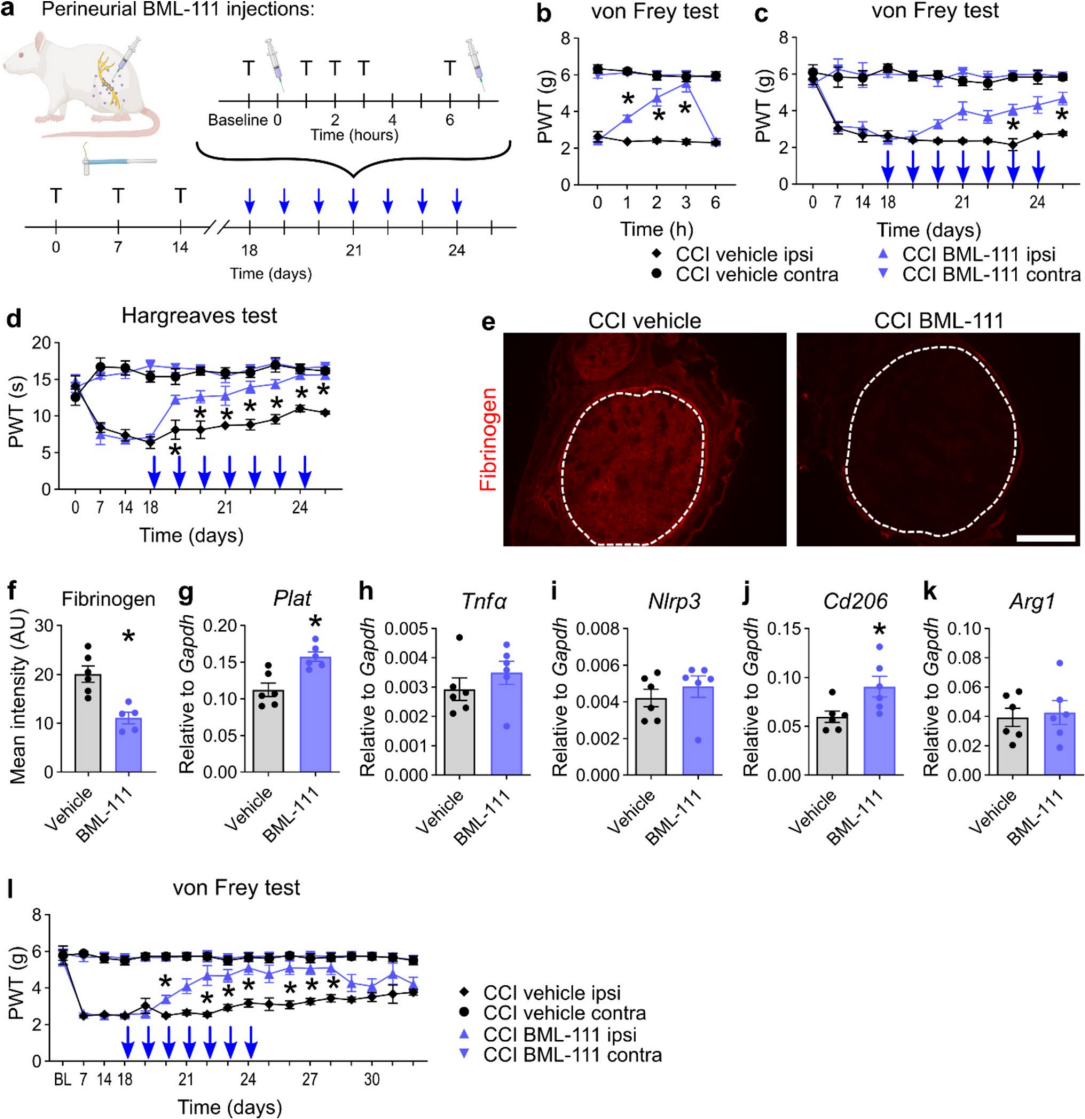
Local application of the Fpr2 agonist BML-111 initiates pain resolution, macrophage polarization and fibrinogen clearance. **a** CCI-operated animals received a perineurial injection of BML-111 or vehicle (PBS). The animals were retested with Hargreaves and von Frey at 1, 2, 3 and 6 h. As soon as reflexive tests at 6 h were finished, the animals received a second injection from day 18 to 24 and were killed at day 25. Blue arrows indicate the daily injection/test programme. T: reflexive testing; syringe: injection. **b** Mechanical allodynia was measured after the first BML-111 injection on day 18 (*n* = 6, two-way repeated-measures ANOVA with Tukey’s multiple comparisons). Paw withdrawal thresholds for **c** mechanical and **d** thermal hypersensitivities of baseline measurements show an accumulative effect of perineurial BML-111 (*n* = 5–6, two-way repeated-measures ANOVA with Tukey’s multiple comparisons). **e, f** Representative images and quantification of fibrinogen immunostaining in the sciatic nerve after BML-111 injections. The dashed lines indicate the endoneurial region. Scale bar: 300 µm (*n* = 6, Student’s t tests with Welch’s correction). **g-k** Relative mRNA expression of *Tnfα*, *Arg1*, *Cd206,* *Nlrp3* and *Plat* after BML-111 injections (*n* = 6, Student’s *t* tests with Welch’s correction). **l** Mechanical allodynia was assessed daily for 1 week after injections were discontinued (*n* = 6, two-way repeated-measures ANOVA with Tukey’s multiple comparisons). *ANOVA* analysis of variance. All data are shown as mean ± SEM, *p < 0.05

Since we identified the AT-LXA4 precursor at the pain resolution switch, we further investigated Fpr2 at 2.5 weeks preceding ‘natural’ pain resolution. To specifically activate Fpr2 in the sciatic nerve, we applied the Fpr2 agonist BML-111 perineurially twice daily for 1 week and tested its analgesic effects using the von Frey and Hargreaves tests (Fig. 6a). The analgesic effect of 500 nmol BML-111 arose from 1 to 3 h after injection and vanished after 6 h (Fig. 6b). Therefore, we decided to administer a second dose after the behavioural test. Cumulative analgesic effects (measured before the first injections of the day) appeared on the 3rd day of injections and gradually increased during the 7-day injection period. While mechanical hypersensitivity significantly improved on day 6, thermal hypersensitivity significantly improved on day 2 and later reached the contralateral paw withdrawal thresholds (Fig. 6c, d). After 1 week of treatment, fibrinogen deposition was reduced in BML-111-treated nerves compared to that in vehicle-treated nerves (Fig. 6e, f). Elevated *Plat* levels in BML-111-treated nerves suggest that fibrinolysis was still in process (Fig. 6g) and not terminated, as observed in CCI animals after 6 weeks (Fig. 3g).

Fpr2 inhibits pro-inflammatory and pro-nociceptive genes such as *Tnfα* and *Nlrp3* and promotes anti-inflammatory macrophages expressing *Arg1* and *Cd206* [7, 74]. In our system, only *Cd206* expression was increased (Fig. 6h–k), highlighting the role of endoneurial fibrinogen. Over the course of 6 weeks, the increased abundance of *Tnfα*, which is known to induce pain, in the CCI nerve compared to sham suggests that *Tnfα* might not be critically involved in pain resolution (SFig. 6). We checked whether *Cd206* was upregulated in the phase of pain resolution; however, *Cd206* levels mirrored the *Plat* levels, while *Cd68* remained stably elevated after 6 weeks (SFig. 6).

To determine whether the activation of Fpr2 induces permanent or irreversible pain relief, we tested the mechanical allodynia of BML-111-administered animals for 1 week after injections. After the last BML-111 treatment, mechanical thresholds remained stable for 3 days and re-approached the levels of vehicle animals, which showed slowly resolving pain (Fig. 6l).

### RvD1 nanoparticles also accelerate resolution in male and female rats

**Fig. 7:**
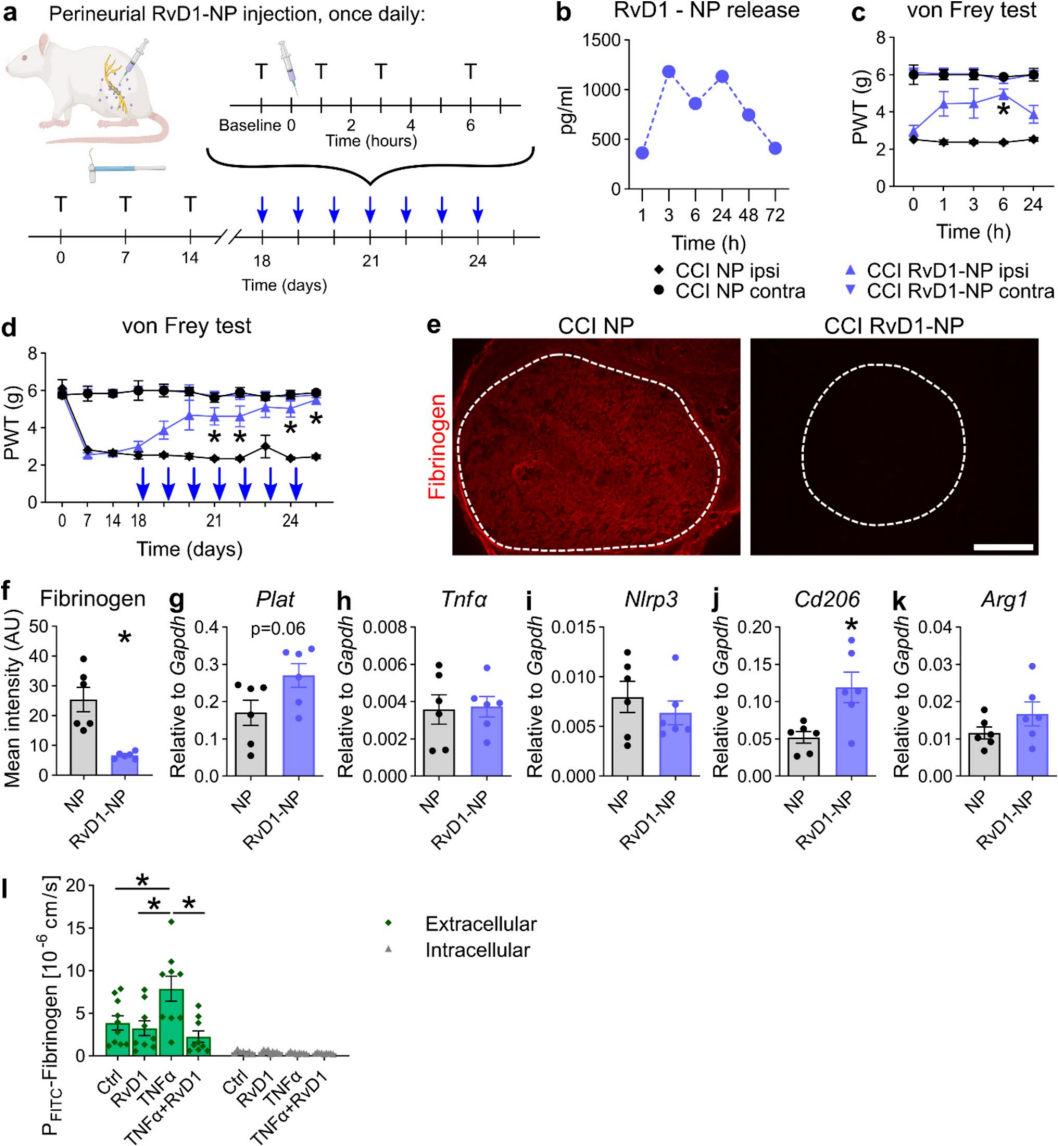
Locally applied RvD1-laden nanoparticles (NPs) induce pain resolution, macrophage polarization and fibrinogen clearance after CCI. **a** Treatment plan for RvD1-NPs in CCI-operated male rats: The animals received a perineurial injection of RvD1-NPs or empty NPs. They were tested at baseline, 1, 2, 3 and 6 h from days 18 to 24, and killed on day 25. Blue arrows indicate daily injection/test programme. *T* reflexive testing; syringe: injection. **b** Estimated amount of released RvD1 from NPs by a dialysis experiment. The NP solution was diluted in PBS and incubated at 37 °C in dialysis tubes. The RvD1 concentration of the dialysates was subsequently determined by LC-MSMS (*n* = 1). **c** Mechanical allodynia was measured after the first RvD1-NP injection on day 18 (*n* = 6, two-way repeated-measures ANOVA with Tukey’s multiple comparisons). **d** Mechanical hypersensitivities measured before the injections during the injection period (*n* = 6, two-way repeated-measures ANOVA with Tukey’s multiple comparisons). **e** Representative images of fibrinogen immunostaining in sciatic nerve cross sections after RvD1-NP injections. The dashed lines indicate the endoneurial region. Scale bar: 300 µm. **f** Quantification of the intensity of fibrinogen immunoreactivity within the endoneurial regions (*n* = 6, Student’s *t* tests with Welch’s correction). **g-k** Relative mRNA expression of *Tnfα*, *Arg1*, *Cd206,* *Nlrp3* and *Plat* after daily RvD1-NP injections (*n* = 6, Student’s *t* tests with Welch’s correction). **l** Fluxes of FITC-labelled fibrinogen through monolayers of primary HDMECs were measured in vitro. Extracellular FITC-fibrinogen fluorescence intensity was assessed to evaluate paracellular barrier integrity, while intracellular FITC-fibrinogen was assessed to evaluate transcellular passage, potentially via endocytosis (*n* = 7–9, one-way ANOVA with multiple comparisons). *NP* empty nanoparticles, *RvD1-NP* RvD1-laden nanoparticles, *ANOVA* analysis of variance, *PBS* phosphate-buffered saline, *CCI* chronic constriction injury, *HDMECs* human dermal microvascular endothelial cells. Scale bar: 300 µm. All data are shown as mean ± SEM, *p < 0.05

Analgesia by BML-111 ceased rapidly, and SPMs were even shorter-lasting. To evaluate SPMs as therapeutic long-lasting Fpr2 agonists, we applied RvD1-laden polymeric nanoparticles (RvD1-NPs, Fig. 7a). The nanoparticles released RvD1 with maximum concentrations between 3 and 24 h (Fig. 7b). Based on these kinetics, RvD1-NPs only needed to be applied once daily to ensure continuous RvD1 supply. RvD1-NPs elicited analgesic effects on mechanical allodynia (Fig. 7c, d). RvD1-NPs caused fibrinogen degradation in the endoneurium and a trend for increased *Plat* (Fig. 7e–g). Similar to BML-111-treated animals, *Tnfα*, *Nlrp3* and *Arg1* levels did not change after RvD1-NP injection, and *Cd206* was upregulated (Fig. 7h–k). Similar effects were observed after RvD1-NP treatment in female rats, suggesting a similar mechanism in male and female rats (SFig. 7).

To assess the effects on the endothelial barrier, we conducted experiments employing HDMECs, which are of endothelial origin and possess typical features that are present in endoneurial vessels as well. Fluxes of FITC-labelled fibrinogen through a monolayer of HDMECs were assessed to study the barrier after challenge with TNF-α and treatment with RvD1. Treatment with TNF-α significantly increased the paracellular permeability for FITC-fibrinogen, while transcellular routes remained unaffected (Fig. 7l). RvD1 was able to completely restore this TNF-α-induced barrier defect (Fig. 7l).

### Fpr2-fibrinogen clearance pathway is inhibited by TAM receptor blocking

**Fig. 8:**
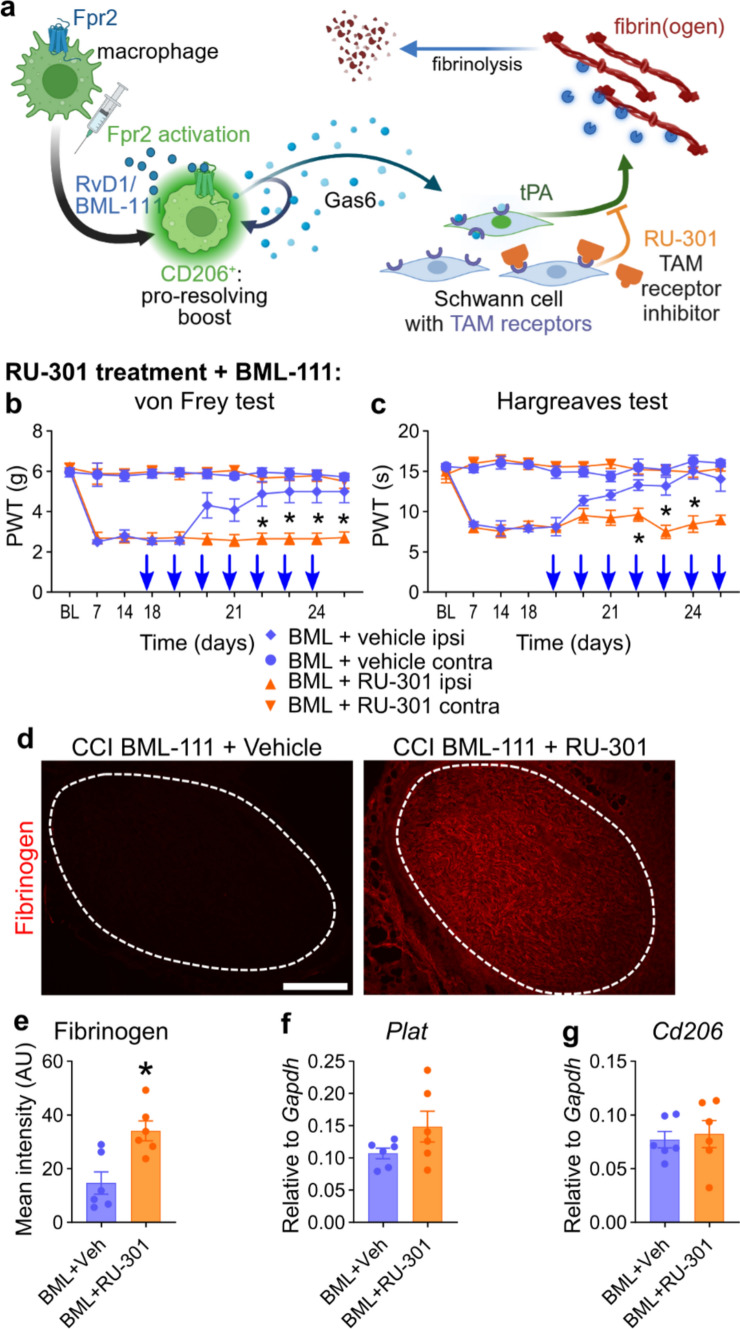
Blocking TAM receptors inhibits Fpr2-mediated pain resolution and fibrinogen clearance. **a** Proposed model of pain relief through Fpr2 activation and involvement of the TAM receptor family of receptor tyrosine kinases (Tyro3, Axl and MerTK) in macrophages and Schwann cells leading to fibrinogen clearance: For details, see text. **b, c** Male Wistar rats received BML-111 injections combined with prior injections of the TAM receptor inhibitor RU-301 for 1 week. Paw withdrawal thresholds for **b** mechanical and **c** thermal hypersensitivities (*n* = 6, two-way repeated-measures ANOVA with Tukey’s multiple comparisons). **d, e** Representative images of fibrinogen immunostaining and quantification within the endoneurial regions in sciatic nerve cross sections after BML-111 with or without RU-301 injections. The dashed lines indicate the endoneurial region (*n* = 6, Student’s *t* tests with Welch’s correction). Scale bar: 300 µm. **f, g** Relative mRNA expression of *Cd206* and *Plat* after 1 week of BML-111 administration with and without TAM blocking

Schwann cells are the main drivers of fibrinogen clearance through tPA secretion [1, 47] but do not express Fpr2. Growth arrest-specific 6 (Gas6), the cognate glycoprotein ligand for TAM receptors secreted by macrophages, is necessary for Schwann cell maturation after nerve injury [61]. Thus, we hypothesized that Fpr2 activation induces Gas6 release from macrophages, which triggers tPA secretion in Schwann cells and leads to fibrinogen clearance (Fig. 8a). In a previously published scRNAseq dataset (Supplementary Methods), we found *Gas6* expression in macrophages and epineurial fibroblasts (SFig. 8a). A lot of macrophages were *Cd206*^+^ anti-inflammatory macrophages, while *Cd80*^+^ pro-inflammatory macrophages were not abundant (SFig. 8a). We further found that *Gas6* expression was more prevalent in *Cd206*^+^ than in *Cd206*^*−*^ macrophages (SFig. 8b, c). In addition, the Gas6 receptors *Axl* and *Mertk* were expressed in Schwann cells (SFig. 8d). Further functional evidence supported the TAM pathway: Co-injection of the TAM inhibitor RU-301 to block TAM signalling before each BML-111 injection inhibited the analgesic effects of BML-111 on mechanical and thermal hypersensitivity (Fig. 8b, c). Under TAM blockade conditions, endoneurial fibrinogen was not cleared although *Plat* levels were not reduced (Fig. 8d–f). *Cd206* was not upregulated (Fig. 8g). We expected that *Cd206* would not be changed by TAM blocking since we hypothesized that the *Cd206* and macrophage polarization increase was mainly caused by Fpr2 stimulation. But TAM receptors also mediate the macrophage phenotype shift [68] and are also necessary for the Fpr2-mediated macrophage phenotype shift. In the scRNAseq dataset, only very few pro-inflammatory *Cd80*^+^ macrophages were present. In our nerve samples, we found many CD206^+^ and basically no CD80^+^ cells throughout 1–6 weeks after CCI (SFig. 9). Instead of a macrophage phenotype shift, we propose a pro-resolving boost of macrophages. These results indicate that TAMs and their ligands are critically involved in pain resolution and fibrinolysis.

## Discussion

In this study, we characterized the pathophysiology of the injured sciatic nerve at the switch towards pain resolution and identified local treatment options for its acceleration. Beyond the known pathways, we included neural barriers, the coagulation system and TAM receptors in this process. Pain resolution and functional recovery were independent processes in both male and female rats. Hyperalgesia was intertwined with enhanced diffusion and failed removal of endoneurial fibrinogen, whereas all other neural barriers were intact or persistently open. Using a translational approach, we further showed that—in line with the findings of the rat model of neuropathy—fibrinogen immunoreactivity was enhanced in nerve biopsies from neuropathic patients with pain in comparison with those without pain. Lipidomic screening of SPMs and their receptors identified Fpr2 ligand precursors at switch points. Fpr2 ligands, such as BML-111 and RvD1-NPs, profoundly boosted fibrinogen clearance and pain resolution beyond immediate application. TAM receptors mediated this signalling cascade through fibrinolysis. In patients with painful polyneuropathies, endoneurial fibrinogen deposition and anti-inflammatory activity were increased, which might indicate similar mechanisms. These novel findings provide new options for the treatment of unresolved neuropathic pain when fibrinogen deposition is prevalent.

The course of thermal and mechanical allodynia after CCI in male and female rats was similar to that previously described [60]. Gait patterns did not completely recover, which might be attributed to residual muscle weakness or coordination deficits and possibly higher sensitivity compared to von Frey in CCI rats [11]. However, CCI rats still reached similar distances and speeds on the voluntary running wheel, suggesting that their overall activity was unaffected.

In our permeability assays, none of the nerve barriers completely recovered with the resolution of pain. The perineurium remained leaky until week 9, which was also reflected by the low expression of tight junction proteins. Similar findings have been previously reported [25]. The open perineurium most likely enabled the diffusion of the perineurally applied substances, which would not be possible in conditions with a sealed perineurium. The perineurial barrier can be transiently opened with hypertonic saline. Simultaneous application allows for the delivery of therapeutics [22].

Interestingly, the capillary barrier remained permeable to the EBA tracer until 6 weeks; however, at this time point, fibrinogen deposition was already cleared from the endoneurium. We postulate that fibrinogen clearance from the endoneurium is necessary for complete recovery of the capillary barrier. Endoneurial fibrinogen drives axonal degeneration and prevents Schwann cell redifferentiation [1, 2, 13], while successful clearance of fibrinogen and myelin debris allows Schwann cell remyelination [2, 47]. In turn, dedifferentiated Schwann cells induce opening of the capillary barrier, as evidenced by immune cell infiltration and EBA tracer extravasation, both of which recover when Schwann cells redifferentiate [42], indicating that barrier closure is dependent on fibrinogen clearance. This could explain the delayed complete recovery of the EBA tracer permeability. The tight junction mRNA expression was in accordance with the results of the permeability assay. However, *Malong *et al*.* showed in a non-injury model that Schwann cells exclusively induce barrier breakdown by increasing transcytosis rates, while tight junctions remain intact [37]. The exact mechanism of leakage remains unknown in our analysis.

Pain resolution coincided with the clearance of endoneurial fibrinogen rather than with inflammation or resealing of capillary vessels for smaller molecules. Possible indirect pro-nociceptive mechanisms of fibrinogen might involve cytokine release from Schwann cells [27, 71] or inhibited anti-nociceptive mechanisms [54] when in contact with fibrinogen. In addition, fibrinogen might directly interact with neuronal axons via toll-like receptor 4, as suggested by *Lim *et al*.* [31]. Macrophages or microglia are activated by fibrinogen through the CD11b/CD18 receptor and induce inflammatory mechanisms in the CNS [48], but to our knowledge, there is no study investigating the effect of fibrinogen on macrophages in the PNS. As fibrinogen is a pro-nociceptive and pro-inflammatory molecule [31], its degradation**,** together with less diffusion, alleviates hypersensitivity.

In the case of fibrinogen deposition, pain relief could also be achieved by targeting fibrinogen. Intravenous recombinant tissue plasminogen activator (rtPA) is used clinically for stroke treatment. To overcome half-life issues and adverse effects such as haemorrhaging, researchers have been developing nanoparticles as carriers for rtPA [8, 14, 16, 28, 36]. One of the proposed advantages of nanoparticles is that they can be developed to not penetrate the blood–brain barrier [36]. Since nanoparticles might be too large to penetrate the open BNB, perineurial injection, as we did, could be considered. Considering the adverse effects of the current rtPA therapy or analgesic drugs, SPMs could serve as promising alternative in pain therapy as they do not induce any analgesic effects in sham models or naïve rodents [26, 64, 70, 73], but adverse effects need to be properly assessed.

In our lipidomic analysis, only 11 out of 34 lipids were detected in the sciatic nerve of CCI or sham animals. SPMs themselves were undetectable. This may be because of nutritional or technical reasons. First, the standard chow is probably not rich in omega-3 acids. Indeed, feeding fish oil drastically increases RvD1 levels [32]. Second, the methods to measure endogenous SPMs and their detectability in the context of their instability and low concentrations are controversial [55]. Exogenously applied SPMs and their analogues have shown pro-resolving effects in different tissue types and conditions in numerous studies, including ours [49, 63]. In fact, the encapsulation of SPMs into nanoparticles seems to be a promising technology for the delivery of anti-inflammatory substances [39, 51].

The effect of Fpr2 activation is most likely multifactorial and reciprocal. In our in vitro barrier model, RvD1 protected the monolayer against fibrinogen flux after TNF-α challenge. But also, TAM signalling and fibrinogen act on endothelial cells [3, 23, 41]. Fibrinogen increases endothelial permeability [45, 46, 66], whereas TAM signalling strengthens barrier integrity [38]. The role of macrophages as guardians of the capillary barrier involves uptake of infiltrating molecules. Under homeostatic conditions, the endothelial barrier in the nerve and dorsal root ganglion allows minimal amounts of blood molecules to pass through, which are taken up by macrophages [34, 37]. TAMs can shift macrophages to a phenotype with pro-resolving properties [68]. In our case, Fpr2 activation might lead to pain resolution through the macrophage phenotype boost, which further activates TAM signalling, leading to sustained *Cd206*-expressing macrophage population, fibrinolysis, plasmin synthesis, debris removal and BNB resealing, alleviating neuroinflammation and thereby neuropathic pain.

The strengths of this study include the diverse array of experimental approaches used to examine the mechanical and thermal hyperalgesia in male and female rats. Furthermore, we examined fully established neuropathic pain and developed a suitable intervention option not only for prevention. We provide evidence that neuropathic pain is triggered by fibrinogen deposition both in an animal model of and in humans with neuropathic pain. We provided a promising treatment option (Fpr2 activation) and an optimized delivery system (RvD1-laden nanoparticles) to resolve neuropathic pain. In addition, we investigated the potential downstream therapeutic targets for therapeutic intervention (TAM receptors and fibrinolysis).

The limitations of this study include missing follow-up investigations of patients. We did not elucidate whether the patients’ pain resolved and lacked healthy controls. Furthermore, the patients were relatively old and did not suffer from nerve injury; therefore, different mechanisms might apply. Human nerve biopsies are rarely performed, and regulations are strictly defined. Therefore, we could not restrict patient inclusion to stringent criteria. We only investigated the CCI model. While similar mechanisms could apply to other models with Wallerian degeneration such as nerve crush or spared nerve injuries, this might not be translatable to other neuropathic pain conditions, such as metabolic or hereditary neuropathies. When transferring our animal results to humans, it needs to be considered that isolated nerve injury is rare and most nerve injuries are accompanied by additional tissue damage, e.g. to muscles or connective tissue. Damage to the surrounding tissue could also induce inflammatory processes that contribute to neuropathic pain. Tissue oedema and inflammation induced by tissue damage might interfere with therapeutic resealing of the barrier. Another limitation was the underlying role of fibrinogen in the maintenance of hypersensitivity. Our study lacks direct proof that fibrinogen causes neuropathic pain in the rat CCI model. Conceptually, direct and conclusive evidence for the role of fibrinogen in neuropathic pain could be obtained by examining fibrinogen-deficient mice [18] or by lipid nanoparticle-encapsulated siRNA-mediated suppression of fibrinogen production [29]. In a translational approach, fibrinogen deposition was elevated in nerve biopsies from patients with painful vs. non-painful neuropathy but, again, this evidence is not conclusive.

The mechanistic pathway presented in this study serves as the basis for the development of therapeutic targets for neuropathic pain. The regulation of macrophage phenotypes and coagulation factors by SPMs and TAM ligands might be a promising approach for inflammatory polyneuropathies in which macrophages accumulate. Tackling the local effects of fully developed neuropathic pain has the potential to resolve chronic neuropathic pain, as it boosts the pain resolution switch. The use of nanoparticles as a delivery system further enables the application of short-lived substances and broadens the molecular toolbox for patient treatment.

## Conclusion

This study links neuropathic pain after nerve injury to capillary leakage in male and female rats. Both pain and diffused endoneurial fibrinogen subsided after local intervention with Fpr2 ligands delivered by nanoparticles. The functional pathway includes an anti-inflammatory/pro-resolving macrophage boost and TAM receptor signalling. This is a novel concept in the field of pain resolution and may shed light on new targets for the treatment of peripheral neuropathies.

## Supplementary Information

Below is the link to the electronic supplementary material.

Supplementary file1 (DOCX 7809 KB)

## Data Availability

Data is provided within the manuscript or supplementary information files.
