# Supplementary material for: TAM receptors mediate the Fpr2-driven pain resolution and fibrinolysis after nerve injury
Source: Acta Neuropathol. 2024 Dec 16;149(1):1. doi: 10.1007/s00401-024-02840-9 (PMC11649718; doi:10.1007/s00401-024-02840-9)
Supplement: Supplementary file 1 — Supplementary file1 (DOCX 7809 KB) [file 401_2024_2840_MOESM1_ESM.docx]

**Supplementary Information**

**Supplementary Material and Methods**

**Immunofluorescence of rat nerve tissue**

For Cldn5 staining, the protocol used in the manuscript was followed. The primary antibodies were mouse anti-claudin-5 (1:100; Thermo Fisher Scientific, Cat# 35-2500), rabbit anti-von Willebrand factor (vWF; 1:100; Agilent, Cat# A0082). For CD206 and CD80 stainings, the sections from the intravenous Evans Blue Albumin experiments were used. Sections were fixed in 4% paraformaldehyde. PBS containing 0.3% Triton X-100, 0.1% Tween20 and 10% donkey serum was used for blocking and permeabilization for 1 h at room temperature. Primary antibodies were rabbit anti-CD206 (1:200; Proteintech, 18704-1-AP) and rabbit anti-CD80 (1:200; bioss, bs-2211R).

**Bulk RNA sequencing data from Schwann cell cultures and nerve tissues:**

RNAseq datasets from nondifferentiated (control) and differentiated (cAMP-stimulated) human Schwann cells were obtained from purified adult human Schwann cell cultures (anonymized, from post-mortem nerve tissues) isolated, expanded, and treated with CPT-cAMP according to established methods (Monje, 2023). Briefly, the cells were harvested from their cell culture dishes by mild trypsinization at time zero (control) and at 1, 2, and, 3 days post-CPT-cAMP stimulation (differentiating treatment). The cell suspensions were collected by centrifugation, snap frozen in dry ice, and stored at -80°C up until processing for RNA isolation and RNAseq analysis, essentially as described in Peng et al., 2020 (Peng et al., 2020). RNAseq datasets from nondifferentiated (control) and differentiated rat Schwann cells were obtained from adult rat Schwann cell cultures (purified, from expanded stocks) subjected to a 3 day stimulation treatment with CPT-cAMP, as reported previously (Camarena et al., 2017).

**Reanalysis of single cell RNA sequencing data of the sciatic nerve after CCI:**

To identify the cell type expressing Fpr2, we reanalysed single cell RNA sequencing (scRNAseq) the experiment by Lovatt and coworkers (Lovatt et al., 2022). Employing the two deepest scRNAseq interrogations from distinct animals of each of the investigated conditions, i.e., of naïve nerves (labelled “Naive”) as well as of nerves 3 d (“CCI3d”), 12 d (“CCI12d”), and respectively 60 d after CCI (“CCI60d”), we focused our re-analysis on the subset of samples (GEO accession number with pool/condition provided in parentheses): GSM6685517 (B5S23b/Naïve), GSM6685518 (B5S24b/Naïve), GSM6685509 (B5S6a/CCId3), GSM6685510 (B5S1b/CCId3), GSM6685496 (B4S1/CCId12), GSM6685520 (B5S3/CCId12), GSM6685521 (B6S31a/CCId60), GSM6685526 (B6S33b/CCId60x). From all of these eight samples, the gene quantifications for each spot (i.e., each single cell) computed by Lovatt et al. were adopted as input to Seurat (v5.0.2, Hoffman P, Satija R, Collins D, Hao Y, Hartman A, Molla G, Butler A, Stuart T (2024). *SeuratObject: Data Structures for Single Cell Data*. R package version 5.0.2, https://github.com/satijalab/seurat-object, <https://satijalab.github.io/seurat-object/>.). Data was normalised by SCTransform() and subsequently combined employing the IntegrateData() functionality of the Seurat package. Clusters of cells with similar transcriptome profiles were delineated employing RunPCA(), FindNeighbors() and respectively FindClusters(). Subsequently, cell types were assigned to the clusters by evaluating manually the respective cell-type specific marker genes reported by Lovatt and coworkers.

**References:**

Camarena, V., Sant, D. W., Huff, T. C., Mustafi, S., Muir, R. K., Aron, A. T., Chang, C. J., Renslo, A. R., Monje, P. V., & Wang, G. (2017). cAMP signaling regulates DNA hydroxymethylation by augmenting the intracellular labile ferrous iron pool. *Elife*, *6*. <https://doi.org/10.7554/eLife.29750>

Lovatt, D., Tamburino, A., Krasowska-Zoladek, A., Sanoja, R., Li, L., Peterson, V., Wang, X., & Uslaner, J. (2022). scRNA-seq generates a molecular map of emerging cell subtypes after sciatic nerve injury in rats. *Communications Biology*, *5*(1), 1105. <https://doi.org/10.1038/s42003-022-03970-0>

Monje, P. V. (2023). Human Schwann Cells in vitro III. Analytical Methods and a Practical Approach for Quality Control. *Bio Protoc*, *13*(19), e4840. <https://doi.org/10.21769/BioProtoc.4840>

Peng, K., Sant, D., Andersen, N., Silvera, R., Camarena, V., Piñero, G., Graham, R., Khan, A., Xu, X. M., Wang, G., & Monje, P. V. (2020). Magnetic separation of peripheral nerve-resident cells underscores key molecular features of human Schwann cells and fibroblasts: an immunochemical and transcriptomics approach. *Sci Rep*, *10*(1), 18433. <https://doi.org/10.1038/s41598-020-74128-3>

**Supplementary Figures**


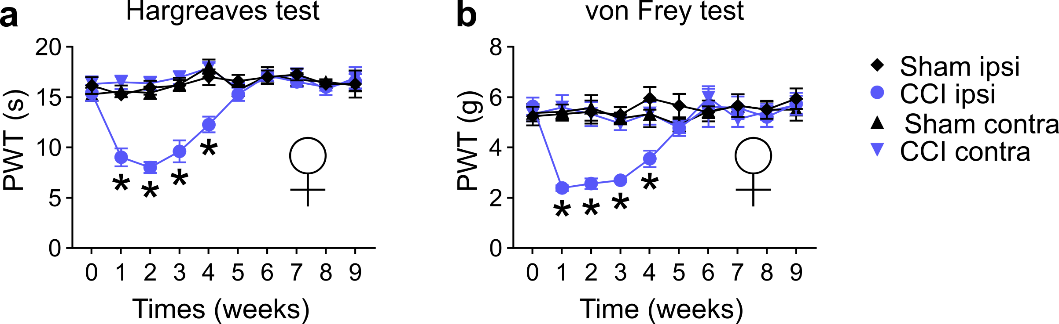


**SFig 1. Neuropathic pain resolves at 6 weeks after nerve injury in female rats.** Female Wistar rats underwent CCI or sham surgery and were examined weekly. **(a)** Thermal and (**b)** mechanical hypersensitivity were assessed (n = 8). Data in graphs are shown as mean ± SEM, * p<0.05. Two-way repeated measures ANOVA with Tukey’s multiple comparison.


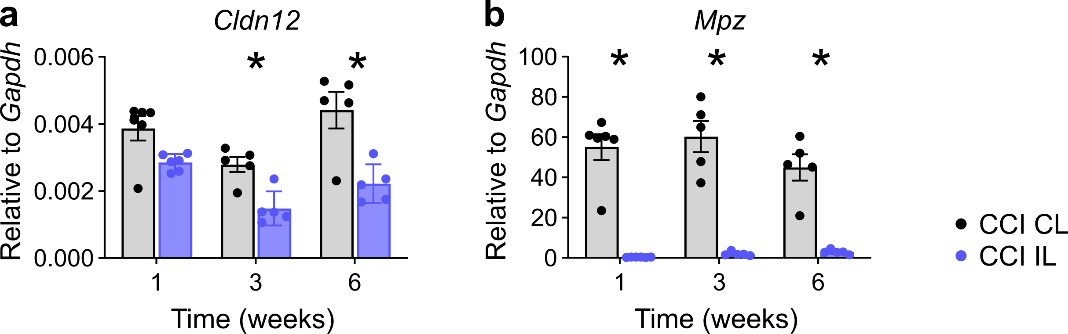


**SFig 2. mRNA expression of *Cldn12* and *Mpz* is reduced after nerve injury.** The ligation part of ipsilateral (IL) and the contralateral (CL) sciatic nerve was assessed (n = 5-6). All data are shown as mean ± SEM, * p<0.05 compared to control at the indicated time points, two-way ANOVA with Šidák’s multiple comparisons.


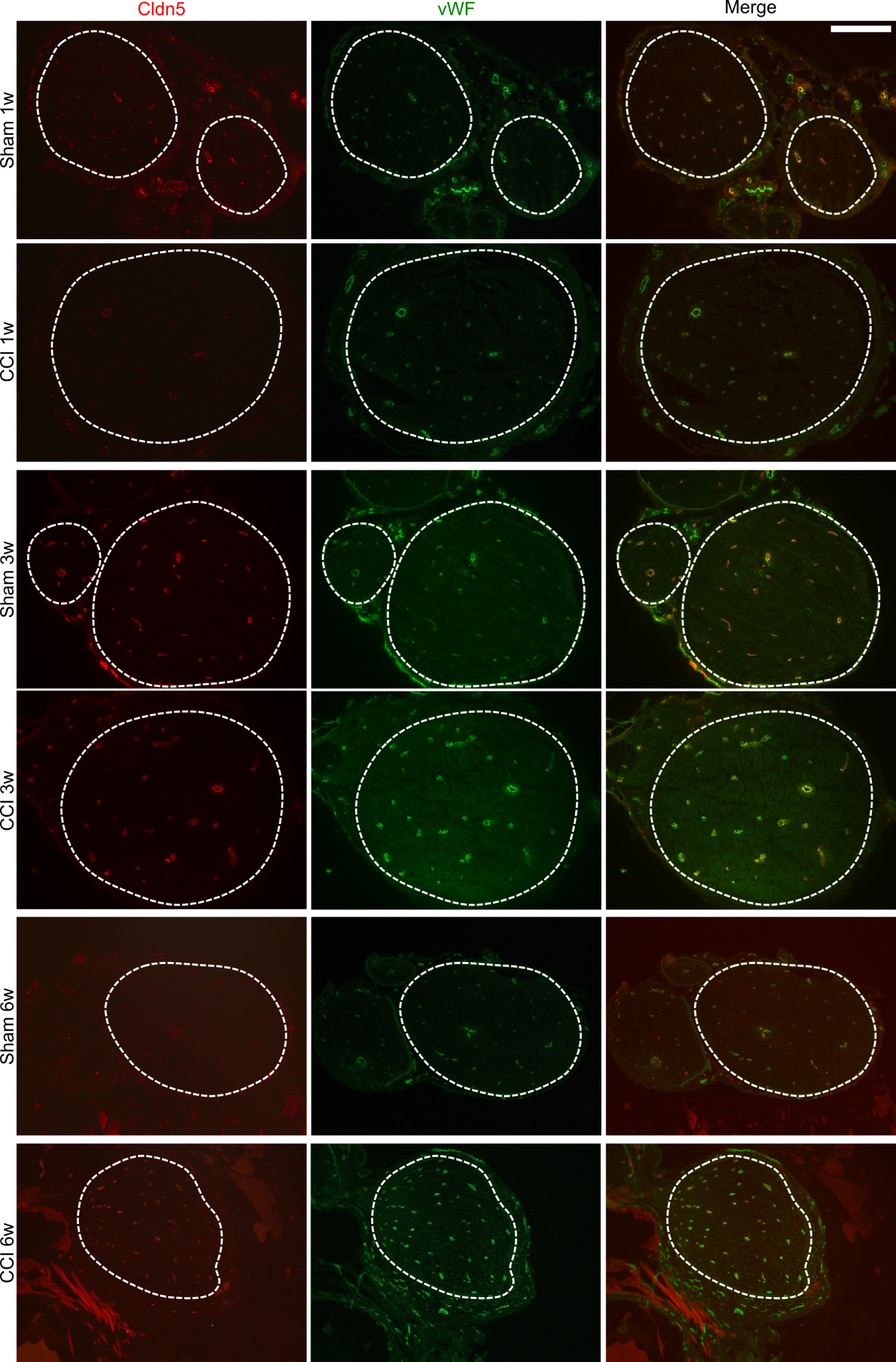
**SFig 3. Exemplary images of Claudin 5 (Cldn5) immunofluorescence in endoneurial vessels after chronic constriction injury (CCI).** In sciatic nerve cross sections, Cldn5 and von-Willebrand factor (vWF) were immuno-labelled after 1, 3, and 6 weeks after CCI and sham surgery. The dashed lines indicate the endoneurial region. Scale bar: 300 µm.


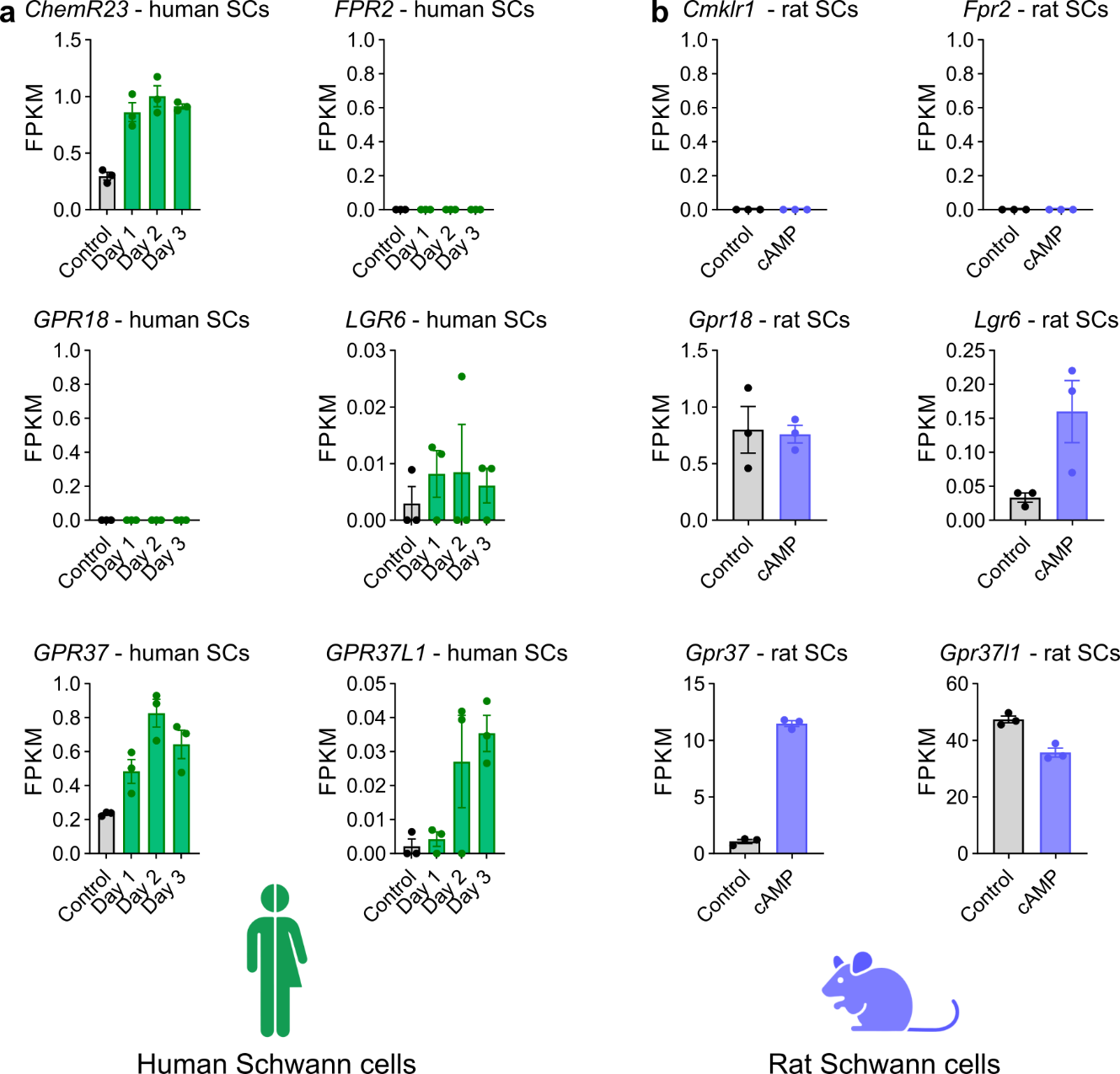


**SFig 4. Different expression patterns of SPM receptors in human and rat Schwann cells *in vitro* after differentiation. (a)** Human and **(b)** rat Schwann cells were stimulated with cAMP *in vitro* to induce differentiation and RNA-sequenced after indicated time points (n = 3). All data are shown as fragments per kilobase of transcript per million mapped reads (FPKM) and mean ± SEM.


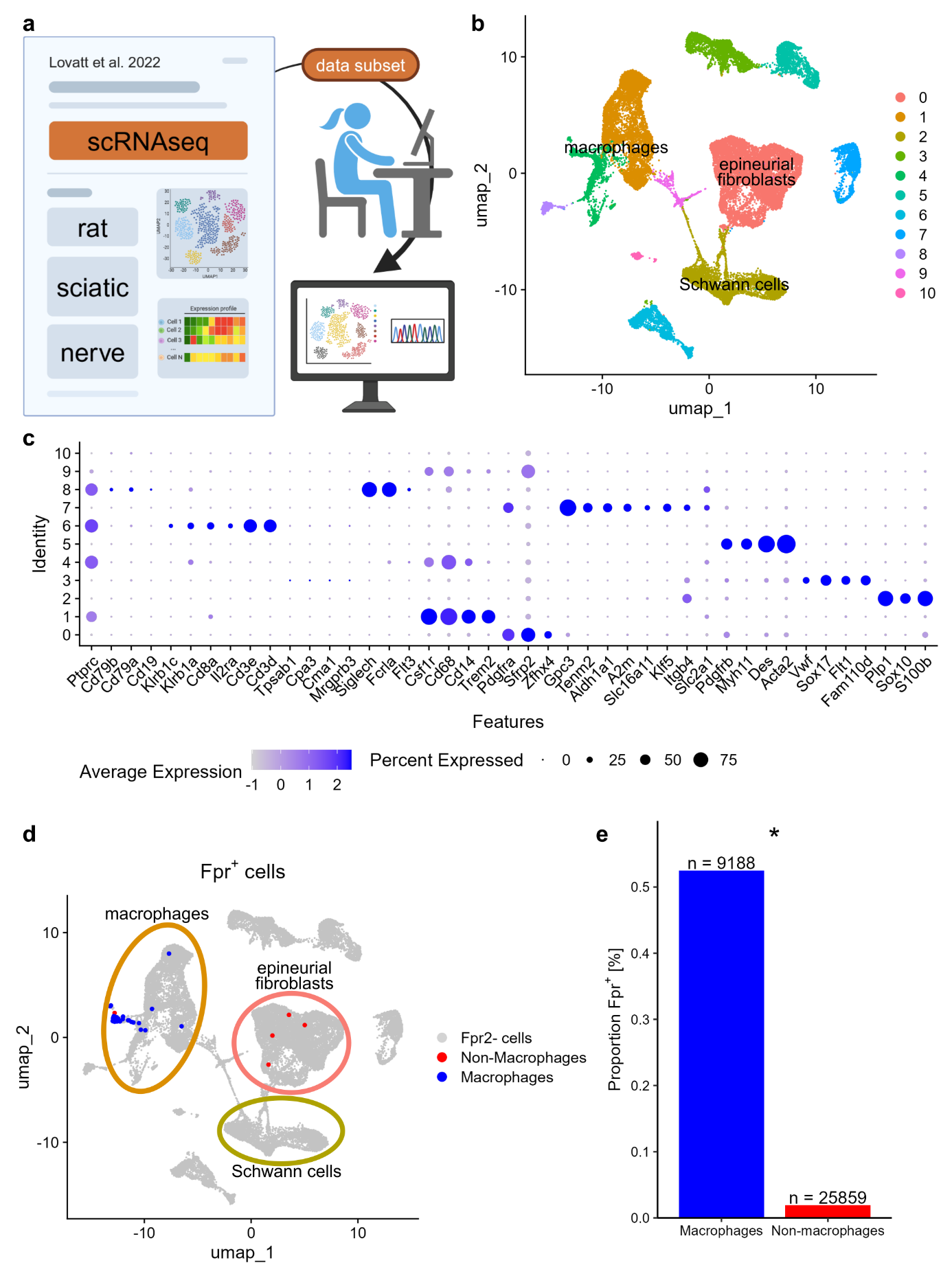
**SFig 5. *Fpr2* enriches in macrophages in sciatic nerve after CCI. (a)** Graphical abstract of our reanalysis (Supplementary Methods) of the experiment by Lovatt et al. (Lovatt et al., 2022): expression quantities for a subset of the previously published single cell RNA sequencing (scRNAseq) dataset of the sciatic nerve after CCI after 3, 12, 60 days, and of the naive nerve was obtained. **(b)** Distribution of the 11 cell cohorts obtained by clustering the 35047 single cells in the experiment, with dimensionality reduced to the first two UMAP dimensions. Subsequent analyses of marker genes (panel c) allowed to assign the cell group with identity 0 to epineurial fibroblasts, the group with identity 2 to Schwann cells, and the cohorts with identity 1 and 4 to macrophages. **(c)** Dotplot visualising the expression of marker genes across major cell types, reproduced from the original publication (Fig. 1c in (Lovatt et al.)). **(d)** *Fpr2^+^* cells within the macrophage clusters (i.e., the cell cohorts with identities 1 and 4 in panel b) are depicted as blue dots while *Fpr2^+^* cells belonging to other clusters are depicted as red dots. **(e)** Proportions of *Fpr2^+^* cells within macrophages and non-macrophages (n= the total number of macrophages/blue bar respectively of non-macrophages/red bar, the asterisk indicates a p-value of <10^-16^ computed by a two-tailed Fisher’s Exact Test for count data).

Lovatt, D., Tamburino, A., Krasowska-Zoladek, A., Sanoja, R., Li, L., Peterson, V., Wang, X., & Uslaner, J. (2022). scRNA-seq generates a molecular map of emerging cell subtypes after sciatic nerve injury in rats. *Communications Biology*, *5*(1), 1105. <https://doi.org/10.1038/s42003-022-03970-0>


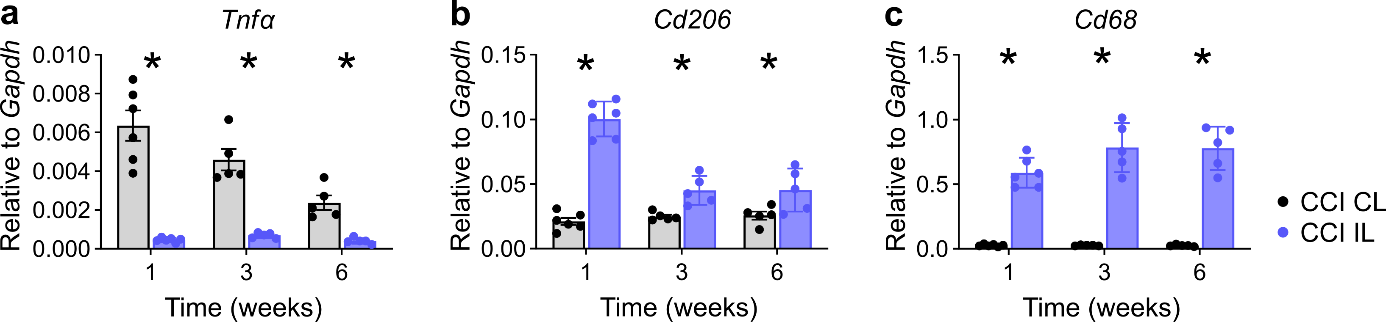


**SFig 6. Nerve injury promotes long-term upregulation of *Tnfα* and macrophage markers.** The ligation part of ipsilateral (IL) and the contralateral (CL) sciatic nerve was assessed (n = 5-6). All data are shown as mean ± SEM, * p<0.05 compared to control at the indicated time points, two-way ANOVA with Šidák’s multiple comparisons.


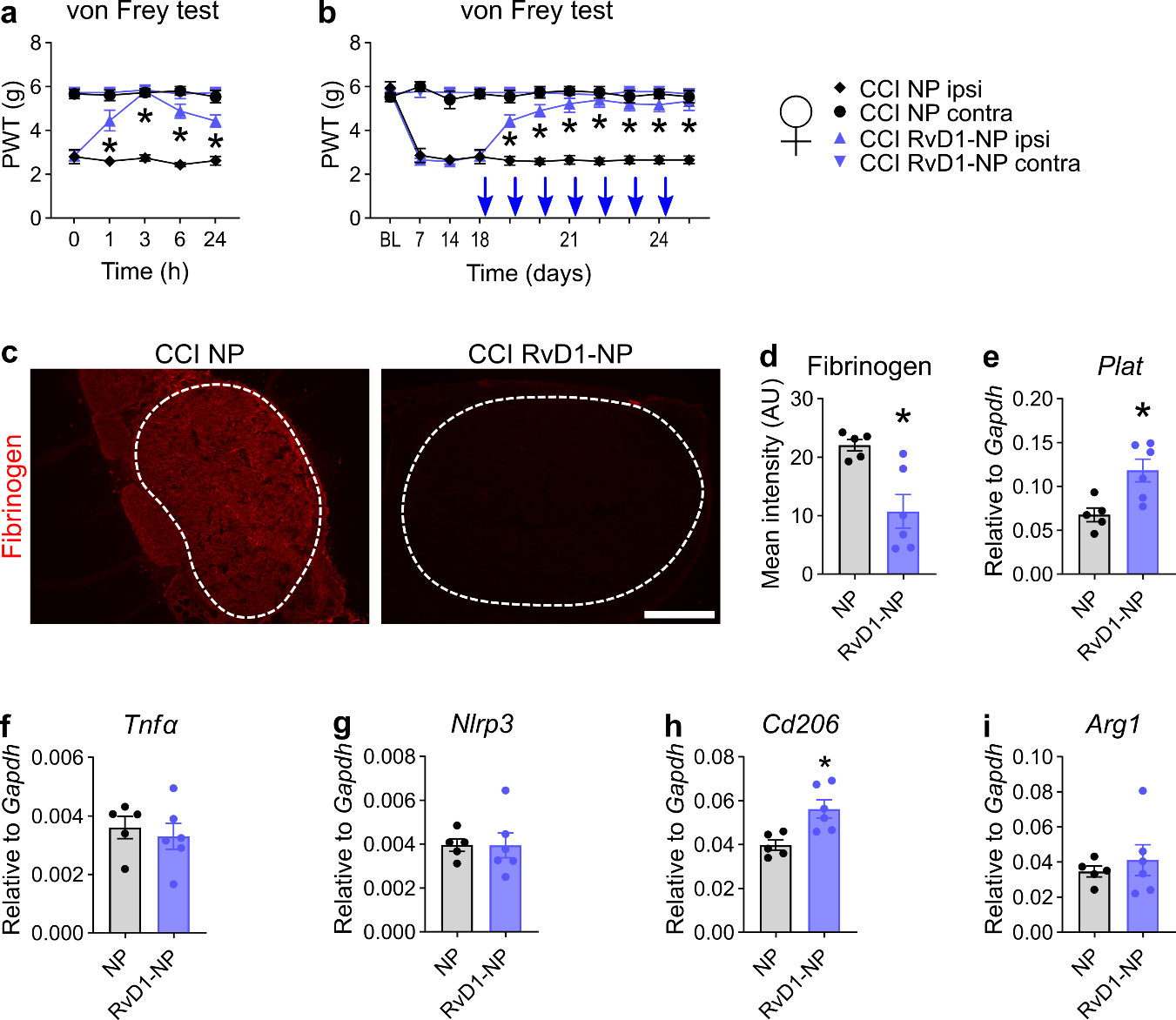
**SFig 7. Similar efficacy and effects for RvD1-laden nanoparticles in female rats.** On day 18 after CCI, animals received a perineurial injection of RvD1-nanoparticles (NPs) or empty NPs. Animals were retested at 1, 2, 3, and 6 h. This daily injection and test program was performed until day 24. Animals were sacrificed for tissue analysis on day 25. **(a)** Mechanical allodynia was measured after the first RvD1-laden nanoparticle injection on day 18 (n = 6, two-way repeated measures ANOVA with Tukey’s multiple comparisons). **(b)** Mechanical hypersensitivities measured before the injections during the injection period (n = 6, two-way repeated measures ANOVA with Tukey’s multiple comparisons). **(c)** Representative images of fibrinogen immunostaining in sciatic nerve cross sections after RvD1-nanoparticle injections. The dashed lines indicate the endoneurial region. Scale bar: 300µm. **(d)** Quantification of the intensity of fibrinogen immunoreactivity within the endoneurial regions (n = 6, Student’s t-tests with Welch’s correction). **(e-i)** Relative mRNA expression of *Plat*, *Tnfα*, *Nlro31*, *Cd206***,** and *Arg1* after daily RvD1-nanoparticle injections (n = 6, Student’s t-tests with Welch’s correction).

NP: empty nanoparticles; RvD1-NP: RvD1-laden nanoparticles. All data are shown as mean ± SEM, *: p<0.05.


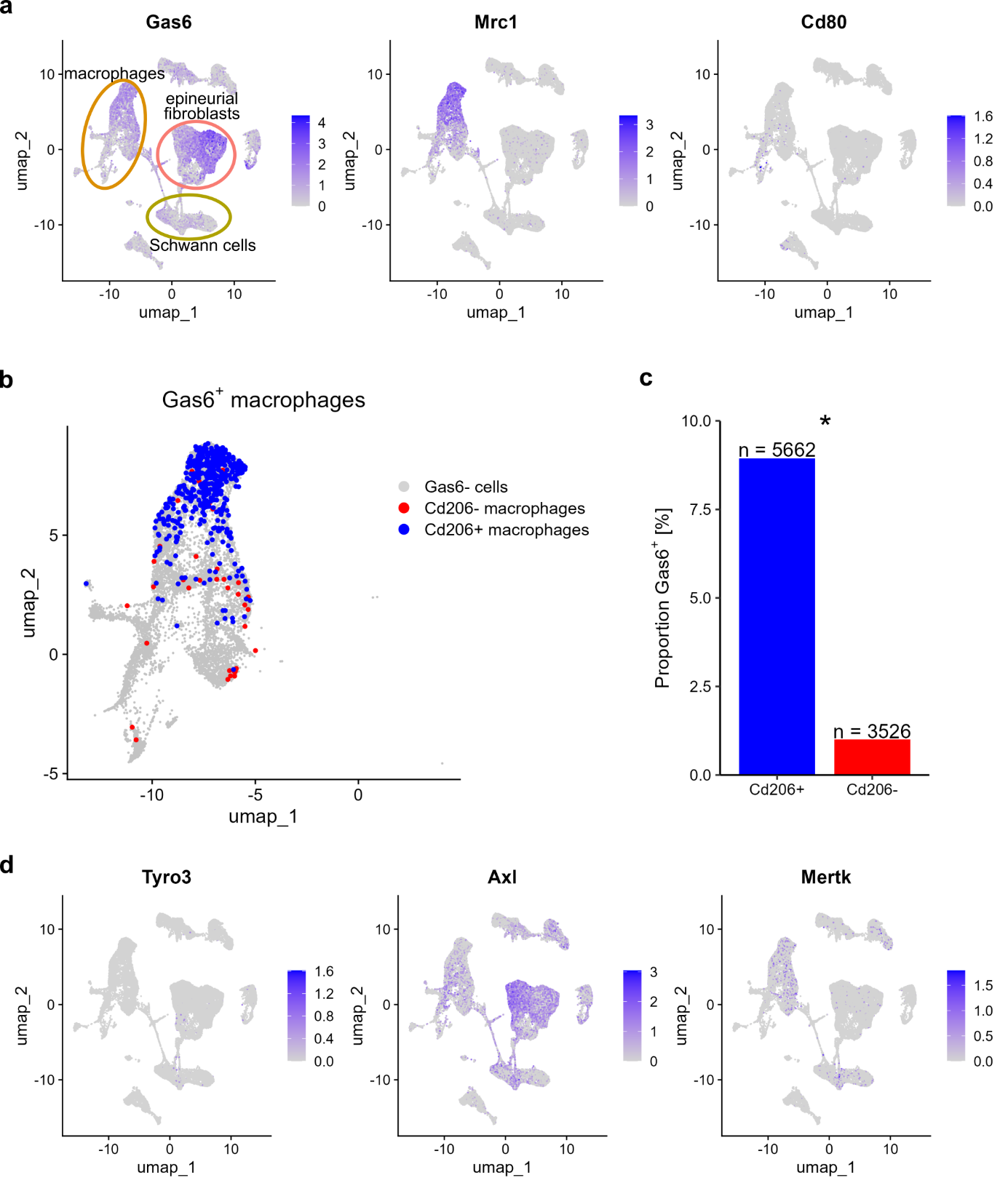


**SFig 8. Especially *Cd206^+^* macrophages and epineurial fibroblasts express *Gas6* in the sciatic nerve after CCI.** All gene expression quantities were obtained from a previously published scRNAseq dataset of naïve sciatic nerves, as well as 3, 12 and 60 days after CCI (Supplementary Methods). **(a)** The expression levels of *Gas6, CD80, and Cd206/Mrc1* are highlighted across the scRNAseq spots arranged according to their UMAP (uniform manifold approximation of projection) coordinates projected to the first two dimensions (SFig. 5). **(b)** The zoomed out macrophage population (i.e., identities 1 and 4) of the UMAP projection depicted in (a), segregated in *Gas6^+^/Cd206^+^* macrophages (i.e. with an expression level of *Gas6* > 2, blue dots), *Gas6^+^/Cd206^-^* macrophages (red dots), and *Gas^-^* macrophages (grey dots). **(c)** Proportions of *Gas6^+^* cells within *Cd206^+^* and *Cd206^-^* macrophages (n= total number of cells, the asterisk indicates a p-value of <10^-16^ calculated by a two-tailed Fisher’s Exact Test for count data. **(d)** Expression levels of the TAM (*Tyro3, Axl, and Mertk*) receptors highlighted across the cells dispersed in their first two UMAP dimensions.

Lovatt, D., Tamburino, A., Krasowska-Zoladek, A., Sanoja, R., Li, L., Peterson, V., Wang, X., & Uslaner, J. (2022). scRNA-seq generates a molecular map of emerging cell subtypes after sciatic nerve injury in rats. *Communications Biology*, *5*(1), 1105. <https://doi.org/10.1038/s42003-022-03970-0>


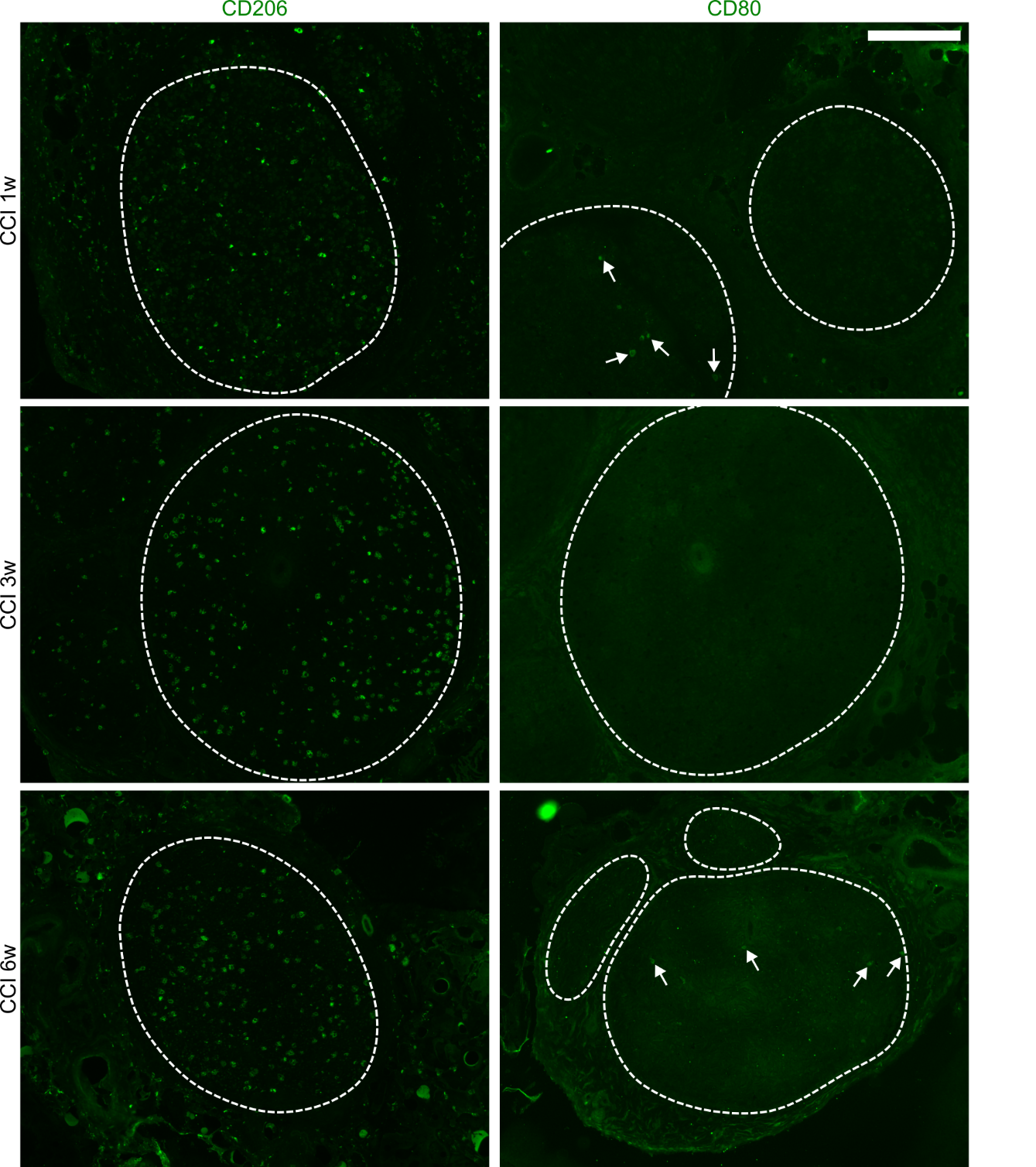


**SFig 9. Numerous CD206^+^ but barely any CD80^+^ macrophages after CCI.** Sciatic nerve cross sections were fluorescently labelled for CD206 or CD80 after 1, 3, and 6 weeks after CCI and sham surgery. The dashed lines indicate the endoneurial region. Arrows indicate CD80^+^ macrophages. Scale bar: 300 µm.
